# Candidate Denisovan fossils identified through gene regulatory phenotyping

**DOI:** 10.1073/pnas.2513968122

**Published:** 2025-08-26

**Authors:** Nadav Mishol, Gadi Herzlinger, Yoel Rak, Uzy Smilanksy, Liran Carmel, David Gokhman

**Affiliations:** ^a^Department of Molecular Genetics, Weizmann Institute of Science, Rehovot 7610001, Israel; ^b^Department of Genetics, The Alexander Silberman Institute of Life Sciences, The Hebrew University of Jerusalem, Jerusalem 91904, Israel; ^c^School of Archaeology and Maritime Cultures, Zinman Institute of Archaeology, University of Haifa, Haifa 3498838, Israel; ^d^Institute of Archaeology, The Hebrew University of Jerusalem, Jerusalem 9190501, Israel; ^e^Department of Physics of Complex Systems, The Weizmann Institute of Science, Rehovot 7610001, Israel; ^f^Sackler Faculty of Medicine, Tel Aviv University, Tel Aviv 69978, Israel

**Keywords:** cranial morphology, Middle Pleistocene crania, genetic phenotyping, Denisovan anatomy

## Abstract

Denisovans are an extinct group of humans whose morphology is mostly unknown. The scarcity of verified Denisovan fossils makes it challenging to study their anatomy, and how well they were adapted to their environment. We previously developed a genetic phenotyping approach to gain insight into Denisovan anatomy by detecting gene regulatory changes that likely altered Denisovan skeletal morphology. Here, we scan the Middle Pleistocene fossil record for crania matching the predicted Denisovan morphology and might therefore be related to Denisovans. We developed quantitative measures to assess both the proportion and extent of matches. These analyses revealed that the East Asian specimens of *Harbin* and *Dali* show an exceptionally high concordance with the Denisovan profile, surpassing all other examined Middle Pleistocene hominin specimens, including Neanderthals. Specifically, 15 out of 18 of *Dali*’s features and 16 out of 18 of *Harbin*’s matched Denisovan predictions. These findings are robust to overall skull size and to correlations between phenotypes. We also found that *Kabwe 1* shows a strong affinity to the Denisovan–Neanderthal clade and might be placed near its root. Our results show that gene regulatory phenotyping may assist in classifying poorly understood specimens.

## Significance

We develop a phenotyping and scanning approach based on gene regulatory differences to identify potential Denisovans in the fossil record. We quantitatively compare genetically predicted phenotypes with morphological measurements and confirm that validated Denisovans are indeed identified through our approach. We apply this method to debated specimens and identify three skulls that are likely closest to Denisovans: *Harbin*, *Dali*, and *Kabwe 1*. Our work lays the foundation for future efforts to infer phenotypes of other extinct hominin groups and to refine the taxonomic classification of the fossil record using genetic data.

Denisovans are an extinct human lineage that likely shared a common ancestor with Neanderthals 390 to 440 thousand years ago (1–4). The first Denisovan finding was reported in 2010 based on DNA extracted from the distal phalanx of a fifth finger (2). Since then, several additional specimens (as well as sediment DNA) have been attributed to Denisovans based on their DNA or protein sequences (1). This collection includes three molars (3, 5), a long bone fragment (3), a partial parietal bone (1, 6), half a mandible with two molars (7), three undiagnostic bone fragments (8) and a rib fragment (9). These remains revealed several aspects of Denisovan morphology, including their large molars (2), a fifth distal phalanx resembling that of anatomically modern humans (AMHs) (10), and an archaic mandibular morphology which includes a robust and relatively low and thick body, without a developed chin (11). Most recently, the *Harbin* cranium (12), and the *Penghu 1* mandible (13), were also molecularly confirmed to belong to Denisovans (14, 15), improving our understanding of Denisovan morphology. Nevertheless, the number of confirmed Denisovan specimens is still low, hindering our ability to study Denisovan adaptations and phenotypic evolution.

The majority of confirmed Denisovan remains have been discovered within the Denisova Cave in Siberia. However, a growing body of evidence indicates that their geographical presence extended further to the east and south. This evidence includes a cranium from Northern China (12), Denisovan sediment DNA and mandible found in the Tibetan plateau (7, 11), a tooth from Laos assumed to be Denisovan based on dental morphology (16), a mandible from Taiwan (13) molecularly confirmed to be a Denisovan, and high admixture rates in populations currently living in East Asia, Southeast Asia, South Asia, and Oceania (1). Thus, it is likely that Denisovans inhabited an extensive geographical range.

Meanwhile, many hominin specimens dated to the Middle and Upper Pleistocene remain poorly classified. The fossil record of these periods is often controversial, and the ability to genetically categorize these remains is limited (17). Consequently, certain specimens have been classified as new provisional lineages [e.g., *H. cepranensis* (18, 19), *H. bodoensis* (20), *H. mabaensis* and *H. daliensis* (21)], while others have been grouped together into broad taxonomic groups such as *H. heidelbergensis* (22), despite their high variability (23). The difficulty in defining and classifying fossils from this period is often referred to as “The Muddle in the Middle” (24).

Importantly, many of these debated specimens have been found in East and Southeast Asia, i.e., in the likely habitat of Denisovans. Notable cranial examples include *Dali* (25), *Jinniushan* (26), *Xuchang 1* (27), *Xujiayao* (28), and *Hualongodng* (29) (for a recent review see ref. 30). Some researchers advocate for their classification as one or more independent species (e.g., *H. juluensis*) (12, 17, 31, 32), while others have suggested that they are eastern representatives of *H. heidelbergensis* (22), or local variants of archaic *H. sapiens* (21, 33). As the academic debate continues, their taxonomy remains unclear, in what was described as a “taxonomic limbo” (21). Following the sequencing of the Denisovan genome, some researchers proposed that some of these specimens might belong to Denisovans (1, 34, 35). Nonetheless, because Denisovans are a lineage defined through their genetics, but these specimens lack genetic or proteomic data, testing whether they belong to Denisovans requires bridging the gap between genetics and morphology. This can be achieved by extracting phenotypic information from the Denisovan genome and comparing it against candidate specimens.

Gene regulatory differences are a key driver of phenotypic evolution and can be highly informative of phenotypic changes between modern and archaic humans (36–40). We have previously developed a method that utilizes gene regulatory data to compare two individuals and discern which one has the higher phenotypic value (e.g., taller stature) (41, 42). This method is based on two key conjectures: *i*) substantial alterations to gene regulation are likely to have a phenotypic effect, and *ii*) down-regulation is expected to affect the phenotype in the same direction as loss-of-function (41). To detect gene regulatory changes between the Denisovan, Neanderthal, and modern human lineages, we leveraged our previously published maps of DNA methylation, which is a key regulatory mark of the genome (43, 44). By linking observations of gene down-regulation with documented phenotypic consequences of gene loss-of-function, we were able to infer morphological profiles. Importantly, we used strict criteria to link genes to phenotypes, filtered out variability caused by confounders such as age, sex, tissue, environment, and disease, and focused on phenotypes with large changes in gene regulation, all pointing in the same phenotypic direction. We tested this approach by reconstructing Neanderthal and chimpanzee profiles and comparing them with their known anatomical features. We found that this method has a prediction accuracy of >85%. Finally, we applied this reconstruction method to the Denisovan lineage, identifying 32 cranial phenotypes that likely distinguished Denisovans from Neanderthals, modern humans, or both (41).

Importantly, the phenotypic predictions comprising the profile are qualitative rather than quantitative—they provide the direction of change, but not its precise magnitude. For instance, while the profile suggests that Denisovans likely had a larger biparietal breadth than both Neanderthals and modern humans, the exact extent of this difference cannot be determined (41). However, unlike quantitative predictions of phenotypic values, predicting the direction of phenotypic differences between individuals can more readily reach high success rates (42).

Here, we leveraged the predicted Denisovan profile to scan the fossil record for Middle Pleistocene crania exhibiting Denisovan-like morphology. We developed approaches to quantify the level of resemblance to the Denisovan profile and revealed that the *Harbin* and *Dali* crania from East Asia closely align with the Denisovan profile, suggesting a strong potential relationship to Denisovans or their close relatives. While the paper was under review, our top match to the Denisovan profile, *Harbin*, was molecularly confirmed as Denisovan, providing positive control and credence to our conclusions. Finally, we found that *Kabwe 1* shows affinity to both the Denisovan and Neanderthal lineage, and our analyses place it close to the root of the Neanderthal–Denisovan split. Our results suggest that gene regulatory phenotyping can enhance our understanding of poorly classified specimens and enable inference of complex phenotypes.

## 1. Methods

### 1.1. Selection of Specimens.

The majority of cranial measurements were directly taken from, or based on, the dataset provided by Ni et al. (12) in Morphobank (45) (project # 3385). The dataset was downloaded on 07/09/2022. Specimens in this dataset were excluded from the analysis if they:

1. Lack a cranium.
2. Predate the Neanderthal–Denisovan split (390 to 440 kya) (1).
3. Belong to a subadult individual.
4. Have fewer than five testable predictions.

**Fig. 1.**
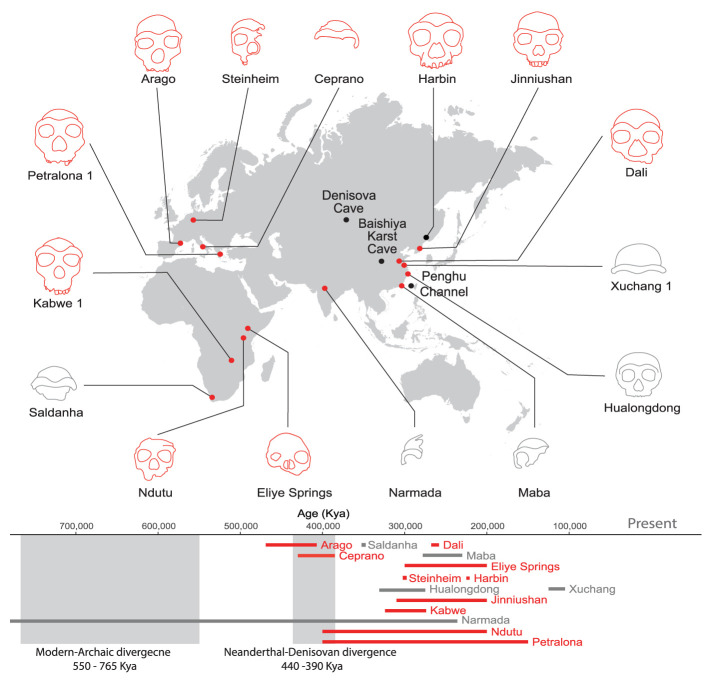
Candidate specimens to be tested against the Denisovan anatomical profile. In red are specimens that were included as test subjects. In gray are crania with fewer than five testable phenotypes, or that belong to subadults. Sites with molecularly confirmed Denisovan remains (Denisova and Baishiya Karst Caves, Penghu Channel, and Harbin) are marked with black dots. Below are the estimated time ranges of the candidate specimens. Crania are not shown to scale.

This resulted in a total of ten test subjects (Fig. 1). Although genetic and archaeological evidence suggests that Denisovans primarily inhabited Eastern Eurasia, we chose not to restrict our search to this region for two key reasons. First, Denisovans are already known to have occupied a wide range of geographical areas, from the Altai Mountains to Laos (1, 2, 16). This suggests the possibility of their presence in additional, yet-to-be-discovered regions. Second, the current genetic evidence for the Denisovan habitat is largely based on introgression patterns in modern populations (1), which primarily reflect Denisovan distribution around the time that AMHs expanded out of Africa. However, the Denisovan habitat in earlier periods may have been different. Finally, including non-Asian specimens-despite their lower likelihood of being Denisovans-could provide insights into their evolutionary proximity to Denisovans. In addition to the group of test subjects, we included three reference groups: *i*) 20 *H. erectus*, *ii*) 18 *H. sapiens*, and *iii*) 15 Neanderthal crania (Dataset S1).

We acknowledge that *H. floresiensis* and *H. luzonensis* share temporal and potential geographic overlap with Denisovans (46, 47). However, they were not included in this study because they were absent from the Morphobank dataset. Despite inhabiting Southeast Asia, their overall morphology and smaller dimensions strongly suggest that they represent distinct non-Denisovan hominin lineages. Similarly, we did not include *H. naledi* specimens in this study due to their established taxonomy, based on their distinct morphology, which places them on a separate hominin lineage from Denisovans (12, 28). *H. naledi* was not used as a reference group in this study, as no available *H. naledi* specimens were available in the dataset.

### 1.2. Cranial Metric Measurements.

**Fig. 2.**
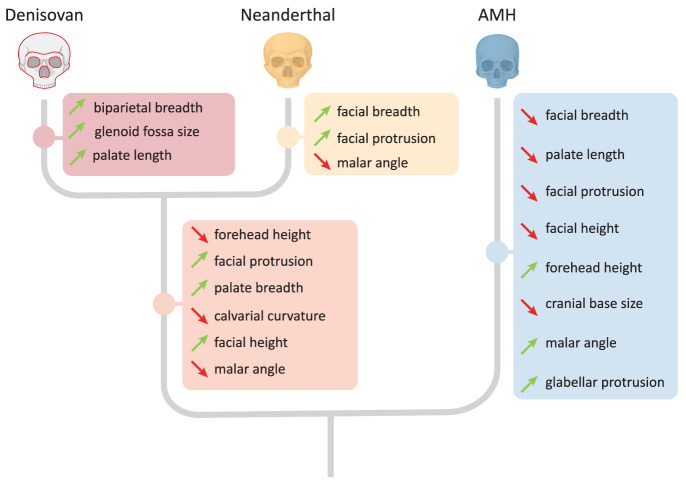
Predicted phenotypic differences between AMHs, Neanderthals, and Denisovans (41). Downward-facing red arrows indicate predicted decreases, and upward-facing green arrows indicate predicted increases. Each phenotype is listed along the lineage where the underlying gene regulatory changes have been observed. Phenotypes may appear multiple times if changes occurred independently in more than one lineage.

**Fig. 3.**
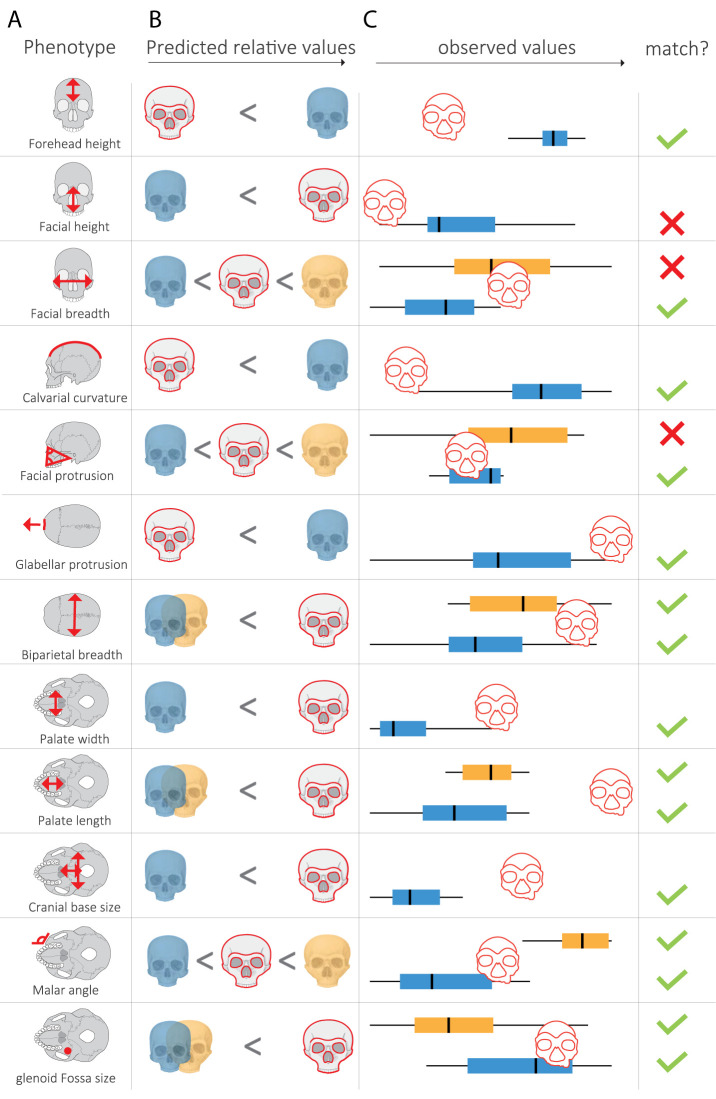
Comparing the morphology of test subjects to the predicted Denisovan morphology. (*A*) The twelve phenotypes used to evaluate the match of each test subject to the predicted Denisovan profile. Each phenotype includes either one or two predictions, based on whether the phenotype in the Denisovan is predicted to differ from AMHs only or from both AMHs and Neanderthals. (*B*) Predicted relative value of each phenotype in Denisovans (red outline), compared to AMHs (blue) and Neanderthals (yellow). (*C*) Box plots showing the distribution of each measurement in AMHs (blue) and Neanderthals (yellow). The red cranium shows the value of this measurement in a test subject, here demonstrated using *Dali*. Check and cross marks show whether or not the *Dali* cranium matches the predicted Denisovan position relative to the other groups.

The reconstructed Denisovan profile included 29 directional predictions, associated with 20 cranial phenotypes in which Denisovans are expected to differ from AMHs, Neanderthals, or both (Dataset S2). Seven of these predictions are associated with nonmorphometric phenotypes (teeth loss timing, teeth eruption timing, skeletal maturation timing, mineralization density), rendering them unquantifiable in the test subjects (Dataset S2). Additional three predictions were not used in our analysis because they were either related to the mandible (pointed chin, mandibular protrusion) or if no matched metric measurement was available (high palate). Last, we also dropped the enamel thickness prediction due to an error in the original reconstruction (see below). In total, the analysis included 18 continuous predictions related to 12 phenotypes (Figs. 2 and 3 *A* and *B*). Predictions were categorized based on the lineage in which they likely arose, i.e., *i*) the AMH lineage, *ii*) the lineage leading from the last common ancestor of AMHs, Neanderthals, and Denisovans to the last common ancestor of Neanderthals and Denisovans, *iii*) the Neanderthal lineage, or *iv*) the Denisovan lineage (Fig. 2).

Each predicted phenotype was associated with a continuous measurement (Dataset S2) based on its description in the Human Phenotype Ontology (HPO) database (48) and other sources of facial morphology [primarily (49)]. For five predictions that did not have a corresponding continuous measurement (related to the phenotypes calvarial curvature, forehead height, glabellar protrusion, and malar flattening), we generated a fitting measurement using available Frankfurt plane-aligned images from Ni et al. (12) (see below). Overall, this resulted in twelve phenotypes, representing 18 predictions of directional phenotypic differences (twelve between Denisovans and AMHs and six between Denisovans and Neanderthals).

Ni et al. (12) provided two matrices in Morphobank: raw measurements (v1.0) and normalized measurements (v1.1). The normalized measurements were generated by dividing the raw measurements by the cube root of the cranial capacity of the respective specimen to account for the effect of body size. We decided to focus on the raw data in the main analyses for several reasons: *i*) many predictions are based on angular measurements, inherently normalizing them for overall size; *ii*) many cranial phenotypes in the original reconstruction suggested higher values in Denisovans. These increases are related to all three dimensions. Such cranial changes could be masked using a dataset normalized by overall size. For example, the dental arch is predicted to elongate in both the mediolateral and anteroposterior dimensions, increasing overall cranial size. Therefore, normalizing by overall size would mask this change and yield misleading comparisons between Denisovans and AMHs; *iii*) The predictions are based on the HPO phenotype definition, that are typically given in raw units; and *iv*) the original estimation of accuracy was based on these absolute values and reached a high accuracy (41). Although absolute measurements are best suited for the gene regulatory phenotyping approach, we also validate the results using several normalization strategies, including the method used by Ni et al. (12), and permutation tests controlling for overall size (see below).

In *SI Appendix*, we provide a detailed description of the predictions and their corresponding measurements (see *SI Appendix*, *Cranial Measurements* and also Dataset S2).

### 1.3. Quantile Estimation.

Each single comparison was carried out by computing the quantile of a particular measurement in a certain test subject with respect to the distribution of this measurement in the reference group (either Neanderthals or AMHs). To this end, let *t* be the value of the measurement in the test subject, and let $t_{1}\leq t_{2}\leq \ldots \leq t_{N}$ be its values in the reference group. The quantile was computed using the following steps: 1) Outliers were removed from the reference group. Values below $Q_{1}-1.5\times \;\text{IQR}$ or above $Q_{3}+1.5\times \;\text{IQR}$ were considered outliers, with the IQR being the interquartile range. 2) The value $t_{i}$ in the reference group was taken as the estimator of the (i−0.5)/N quantile, $t_{i}={\hat{q}}_{(i-0.5)/N}$. The quantile of tied values was taken as the mean of the respective quantiles (the parameter “ties” was set to “mean”). 3) The remaining quantiles were linearly interpolated using the approxfun function in R, to create a cumulative distribution function (CDF). 4) the percentile of *t* was estimated based on the generated CDF.

### 1.4. Phenotypic Distance.

Based on the estimated quantile of a measurement with respect to a reference group (Neanderthals or AMHs), we defined a “phenotypic distance” that provides a normalized score for the distance of this measurement in the test subject from the reference group. This distance lies in the range (−1,1), with positive values reflecting agreement with the prediction in Denisovans, and negative values reflecting disagreement. To compute this distance, quantiles should first be centered around 0 by subtracting 0.5. Then, the predicted direction of phenotypic change is accounted for by flipping the sign if the measurement in Denisovans is predicted to be lower than in the reference group. Finally, the distance is scaled to (−1,1) using multiplication by two. Let *s* be +1 if the measurement is predicted to be higher in Denisovans, and −1 if it is the other way around. Let *q* be the quantile of the measurement in a specific test subject with respect to the reference group. Then, the phenotypic distance would be

$$D=2s\left(q-\frac{1}{2}\right).$$

### 1.5. Scoring the Match Between a Test Subject and the Denisovan Profile.

Let $d_{1},d_{2},\ldots ,d_{k}$ be the phenotypic distances of *k* different measurements obtained for a particular test subject. Based on these distances, we scored how closely the test subject resembles the predicted Denisovan profile using two approaches. First, we consider a measurement to be compatible with the prediction if the corresponding phenotypic distance is greater than zero. Given that under the null hypothesis we have equal chances for a positive and a negative phenotypic distance, we used a one-tailed binomial test to test whether the number of positive values is significantly greater than expected by chance. The binomial test was carried out using the “binom.test” function in R with continuity correction (50, 51). Second, we used the one-tailed Wilcoxon test to check whether the median of the phenotypic distances significantly deviates from zero. This was implemented using the wilcoxsign_test function from the R “coin” package (52). We used this function instead of wilcox.test function since its implementation of the test accepts tied values in the dataset. The *P*-values generated by each of the two tests were then corrected for multiple comparisons using the Benjamini–Hochberg procedure (53). The multiple comparisons correction was performed only for test subjects, as all other specimens were not hypothesized to potentially belong to Denisovans or their close relatives, and therefore cannot be considered discoveries.

The phenotypic features examined in this work are correlated, which violates the assumption of both statistical tests. Hence, we do not assign a statistical meaning to the *P*-values, but rather treat them as scores reflecting the relative similarity of each specimen to the predicted Denisovan profile (see next section for a test that accounts for the correlation between features). The binomial score is based on the binomial test *P*-value, while the Wilcoxon score is based on the Wilcoxon test *P*-value. The scores were calculated from the *P*-values as

$$S=-\log_{10}\left(P\right).$$

### 1.6. Permutations.

A permutation test was applied in order to validate the fact that the measurements we used in the actual analysis are significantly more informative than a random set of measurements. Each permutation was generated by sampling random measurements from the pool of available continuous measurements in the Morphobank dataset of Ni et al. (12). For consistency, the measurements generated for the current work were included in the pool as well (these include calvarial curvature, forehead height, glabellar curvature, cranial base area, malar flattening, glenoid fossa size, and facial protrusion). For each specimen, the original measurements were replaced by other randomly sampled measurements, which were then used to score the resemblance of the test subject to the predicted Denisovan profile.

To avoid potential biases, the permutations were designed to conserve all relevant properties of the original analysis. First, the number of testable predictions of each test subject was maintained across all permutations. Notably, the number of testable predictions differs across specimens, possibly due to differential levels of preservation. For example, the *Petralona 1* cranium is highly preserved and has all 18 testable predictions. In contrast, *Ndutu* has only 9 testable predictions, due to poorer preservation.

Second, the number of predictions tested against each reference group (AMHs, Neanderthals) was kept fixed for each test subject. For example, *Petralona 1* has twelve predictions tested against AMHs, and six that were tested against Neanderthals. These numbers were retained in all *Petralona 1* permutations.

Third, the ratio of linear to nonlinear (i.e., angles or ratios) predictions in each permutation was equal to the ratio in the original predictions. For example, *Dali* has 14 linear predictions and four nonlinear predictions, these numbers were retained in all permutations of *Dali*.

Last, this work is based on predictions of the directionality of phenotypic changes in Denisovans compared to a reference group. However, the direction is arbitrary, as it is based on the way the measurement is defined. For example, a measurement defined as forehead height is predicted to be smaller in Denisovans compared to AMHs. However, were we to define the measurement as forehead shortness, this measurement would have been predicted to be larger in Denisovans. In order to account for this when carrying out the permutations and to maintain the relationships between different measurements, we redefined the directionality of each measurement based on its correlation with biorbital breadth (EKB). Measurements with a positive correlation with EKB (*N* = 118) were considered to have positive directionality, while measurements with a negative correlation (*N* = 24) were considered to have negative directionality. Most measurements were positively correlated with EKB, as expected given its correlation with overall size.

For each specimen, N=10,000 permutations were performed. A combined statistic that takes into account the Wilcoxon score and the binomial score $S_{\text{obs}}=\sqrt{S_{\text{binom}}^{2}+S_{\text{wilcoxon}}^{2}}$ was computed and compared to permuted combined scores to produce the permutation *P*-value: $P=\frac{1}{N}\sum_{i=1}^{N}\left(S_{\text{obs}}\geq S_{i}\right)$. *P*-values were assigned based on the fraction of permutations in which the random match exceeds the observed one. *P*-values for all test subjects with at least five predictions were corrected for multiple comparisons using the Benjamini–Hochberg procedure (53).

### 1.7. Scoring of Denisovan–Neanderthal Differentiation.

Given the taxonomic proximity of Neanderthals and Denisovans, we wished to further examine how well we differentiate these two lineages by repeating the analysis only on predictions that distinguish between them, i.e., predictions that are derived in either Denisovans or Neanderthals. Phenotypes relevant to this analysis were parietal breadth (wider in Denisovans), maxilloalveolar length (longer in Denisovans), facial breadth (wider in Neanderthals), facial protrusion (more protruding in Neanderthals), glenoid fossa size (greater in Denisovans), and malar flattening (more flattened in Neanderthals) (Fig. 3). In this analysis, we examined *Harbin*, *Dali*, and *Kabwe 1*–the three top-scoring test subjects, which were the only specimens to pass the permutation test. We compared them to all Neanderthals, using only specimens with at least five testable predictions.

To further help distinguish between the Denisovan and Neanderthal sister groups and to maximize the use of known Denisovan phenotypes, we added to the analysis molar crown area, a key phenotype known to separate between Neanderthals and Denisovans. Denisovan upper molars [specifically *Denisova 4* (2) and *Denisova 8* (5)] were described as having a crown size well beyond the range of both Neanderthals and AMHs (2, 5, 6, 54, 55). We calculated the molar crown area using the mesiodistal length and buccolingual width of the second molar ($M^{2}$). Since it is unknown if the Denisovan molar was $M^{2}$ or $M^{3}$, we used the more strict $M^{2}$ data in this analysis (since AMH and Neanderthal $M^{2}$ usually have a greater crown size than $M^{3}$). It should be noted that *Harbin* exhibits $M^{3}$ agenesis or at least a very small $M^{3}$, and *Dali* does not have any preserved teeth. Overall, up to seven phenotypes were used to differentiate Denisovans and Neanderthals. Repeating the analysis without molar crown size resulted in *Dali*, *Harbin* and *Kabwe 1* falling within the 97th, 91st, and 69th percentile of the Neanderthal distribution, respectively.

### 1.8. Principal Component Analysis.

Since most crania used in this study are incomplete, many measurements were missing. To compute PCA, missing data had to be imputed. To this end, we used the “missMDA” package (56). This package imputes missing data by iterative PCA, which takes into account the correlation between variables and similarity between samples. Values were imputed using the function “imputePCA” with default parameters: scaled data and two dimensions.

To avoid overimputation of the data, we first filtered out measurements with over 20% missing values (i.e., kept only measurements where 80% or more of the specimens had data), and then specimens with more than 15% missing values. These values were selected in order to keep a maximum number of specimens while still keeping imputation low. We also computed a second PCA in which we first applied the 15% specimen filtering and only then the 20% measurements filtering, in order to try to retain more measurements (*SI Appendix*, Fig. S2).

In addition to these PCA maps, which were computed based on continuous measurements, we computed another PCA based on nonlinear measurements (e.g., angles, ratio) (*SI Appendix*, Fig. S3). This was done in order to test the potential effect of overall size biases on the clustering. Since there are fewer nonmetric measurements in the dataset, we used a slightly more permissive threshold (30% instead of 20%) for maximum missing values per measurement. In this approach, the overall number of imputed values per specimen remained the same, although fewer specimens passed the filtering.

All PCA maps were computed based on the individuals of the reference groups. The test subjects were then projected onto this plane to produce the final plot.

### 1.9. Xiahe 1 and Penghu 1 Mandibular Analysis.

In our previous work (41), we reported that our reconstruction correctly predicted seven out of eight mandibular phenotypes in *Xiahe 1* (11). Here, we repeated this analysis for *Xiahe 1* but modified it to match the logic of the current work in the sense that we considered only predicted differences and excluded traits where no difference is expected. Moreover, we conducted the same analysis for *Penghu 1*, following the report of its potential affiliation with Denisovans. To this end, we used the mandibular data provided in (11, 13, 57).

Five measurements, associated with six predictions, were examined in the *Xiahe 1* and/or *Penghu 1* mandibles: *i*) superior mandibular length (mandibular prognathism prediction), *ii*) bicanine breadth (anterior mandibular width prediction), *iii*) Symphyseal height (anterior mandibular height prediction), *iv*) condylar head surface area (condylar size prediction), and *v*) dental arch length (dental arch length prediction).

**Fig. 4.**
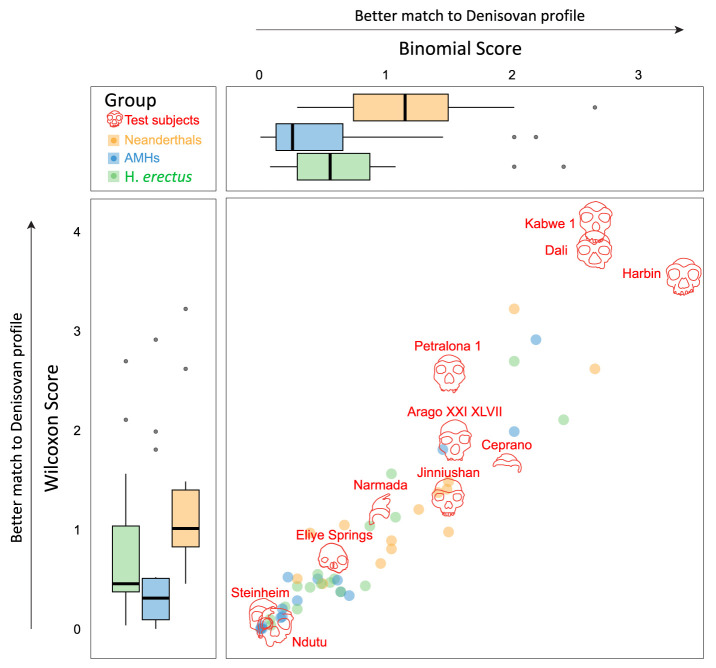
Testing various specimens against the predicted Denisovan profile. Each specimen was tested for up to 18 predictions expected to distinguish Denisovans from AMHs and/or Neanderthals. The scatter plot shows the match of each specimen to the Denisovan profile using two scores—binomial (x-axis) and Wilcoxon (y-axis). Boxplots show the distributions of scores for the reference groups, serving as controls.

The superior mandibular length of *Penghu 1* was previously shown to be longer than the median of Early *H. sapiens*, in accordance with our prediction (13), figure 4a]. This measurement is not available for *Xiahe 1*. Bicanine alveolar length was also shown to be longer in *Penghu 1* and *Xiahe 1* than the median of Early *H. sapiens* (57), figure S26F], in accordance with our predictions. The symphyseal height of *Xiahe 1* is longer than that of Early H. sapiens (57), figure S26F], in accordance with our prediction. However, the same measurement for *Penghu 1* is shorter than the median of Early *H. sapiens*, thus contradicting our prediction. The condylar head of *Penghu 1* was reported to be short mediolateraly, but also anteroposteriorly wide (13). Using the provided superior image of the mandible [figure 1 in (57)], and the provided mediolateral dimension of the condyle, we estimated the condylar head area (approximated as an ellipse) to be 189.34 mm^2^. This area is larger than the equivalent condylar areas in modern male samples, which exhibit a mean area of 133.63 mm^2^ (58). Thus, the condylar head area of *Penghu 1* is in accordance with our prediction. The condyle of *Xiahe 1* is missing. In addition, we were not able to obtain the equivalent measurements for Neanderthal condyles. Last, the dental arcade length of *Xiahe 1* is provided in (11). Comparable AMH values were computed as a weighted mean of the Early *H. sapiens*, Late *H. sapiens*, and Asian *H. sapiens* groups (11). Neanderthal values represent the weighted mean of Asian Neanderthals and European Neanderthals. The dental arcade length of *Xiahe 1* (55.7 mm) is longer than those of both AMHs (52.58 mm) and Neanderthals (54.78 mm). The dental arcade length of *Penghu 1* is not directly reported but can be inferred upon indirectly. First, the alveolar arcade index (M2) of *Penghu 1* is known to be higher than in Early *H. sapiens* and Neanderthals (13), figure 4o]. Second, the incisal and premolar portion of the *Penghu 1* dental arcade are wider than in both Early *H. sapiens* and Neanderthals (13), figure 4o-p]. Therefore, the dental arcade of *Penghu 1* is necessarily longer than both H. sapiens and Neanderthals, matching our predictions.

Tsutaya et al. (57) carried out a similar analysis specifically for the *Penghu 1* mandible. However, they reached a different conclusion due to deviations from the pipeline described above. Specifically, *i*) the use of phenotypes that do not accurately depict the original HPO phenotype (e.g., not including the anterior–posterior dimension of the mandibular condyle in the analysis of condylar size); *ii*) interpretation of unobserved changes as predictions of no change, whereas unobserved changes usually result from contradicting or insufficient information to determine the direction of change; and *iii*) the use of size-normalized measurements which, as we explained above, are less appropriate in our analysis.

### 1.10. Map.

The background world map used in Fig. 1 is based on the world map by PT Northern Lights Production, used under GPLv.2 license. Available at: https://mapsvg.com/.

## 2. Results

### 2.1. Denisovan Specimens Validate Denisovan Genetic Phenotyping.

Morphologically informative Denisovan specimens can be used to further evaluate the accuracy of the Denisovan profile suggested in Gokhman et al. (41). These include *Xiahe 1* (11), the Denisova 3 distal phalanx (10), several molars (3, 5), and *Penghu 1*, which was reported as a likely Denisovan while this paper was in review (57).

Six morphological predictions can be evaluated using the two available Denisovan mandibles (11, 57). Compared to AMHs, both mandibles exhibit the predicted greater prognathism, and wider anterior. Both mandibles also show the predicted longer dental arch compared to both AMHs and Neanderthals. The profile also predicts Denisovans to have a greater symphyseal height compared to AMHs, which is confirmed in *Xiahe 1*, but not in *Penghu 1*. Last, *Penghu 1* exhibits the predicted larger condylar head size compared to AMHs (this prediction cannot be tested in *Xiahe 1*, which lacks a condylar head). In total, 90% of our mandibular predictions were confirmed, with five out of six available predictions confirmed in *Penghu 1*, and four out of four in *Xiahe 1* (*Methods*). Nevertheless, the number of testable mandibular predictions is small, and further specimens are needed to more accurately assess how well our profile reflects Denisovan morphology.

The Denisova 3 distal phalanx (10) could potentially be used to evaluate the prediction of reduced tapering of the finger from the base of the proximal phalanx to the fingertip in Denisovans compared to AMHs. However, this assessment requires proximal and middle phalanges, which have not yet been discovered, leaving the accuracy of this prediction to be determined. Finally, despite the availability of Denisovan molars (3, 5), our predictions of Denisovan dentition pertain to the timing of tooth eruption and loss, which cannot be determined using available specimens (see *Cranial Metric Measurements* in *Methods* for *tooth enamel*).

The *Harbin* cranium, confirmed to be a Denisovan while this work was under review, (14, 15) also shows strong concordance with the predicted Denisovan profile, matching 16 of 18 traits (see below).

In total, 25 out of 28 (89%) predictions by the original profile have now been validated by subsequently discovered specimens. This level of concordance aligns closely with the estimated precision of our gene regulatory phenotyping approach (>85%), tested by applying it to Neanderthal and chimpanzee datasets (41).

### 2.2. Test Subjects and Measurements.

Given the accuracy of the genetic phenotyping approach, we turned to scan the fossil record for specimens matching the reconstructed Denisovan profile. We started by generating a set of candidate fossils, which we denote test subjects. This set was constructed using several criteria. First, we only examined specimens whose dating partially or completely postdates the emergence of the Denisovan lineage, i.e., the estimated Denisovan–Neanderthal split [390 to 440 kya, (1)]. Second, non-Denisovan specimens whose taxonomy is well defined (e.g., Neanderthals, *H. erectus*, and AMHs) were not included in the set of test subjects but rather as reference groups. Due to the ongoing debate about the monophyly and taxonomy of *H. heidelbergensis* specimens (59, 60), as well as their phylogenetic proximity to Denisovans, which led some to suggest that some *H. heidelbergensis* may in fact be Denisovans (34), these specimens were included among the test subjects. Third, to allow for enough power in the analysis of each specimen, and because the predicted Denisovan profile contains many cranial features, we focused on sufficiently complete crania, containing at least five testable predictions. Fourth, we did not include subadult specimens, as they could not be directly compared to the adult specimens of the reference groups. Finally, to minimize biases and to account for the possibility that the Denisovan habitat extended beyond Eastern Eurasia, we did not filter out specimens based on their location. Altogether, the set of well-preserved Middle Pleistocene cranial test subjects included ten specimens (Fig. 1). These test subjects lack genetic characterization, display substantial morphological diversity, and their phylogenetic relationship to known hominin groups is uncertain or subject to debate.

To assess which specimens match the predicted Denisovan morphology, we tested each cranium against 18 predictions related to 12 phenotypes (41). In all 12, Denisovans were predicted to differ from AMHs, and in six of them, Denisovans were also predicted to differ from Neanderthals (Figs. 2 and 3 *A* and *B*). Thus, depending on the degree of cranial preservation, up to 18 comparisons were carried out for each test subject. Also, 13 out of the 18 predictions could be directly matched against measurements in the Morphobank dataset (12). The other five (related to the phenotypes calvarial curvature, forehead height, glabellar protrusion, and malar flattening), had no matching quantitative information in the Morphobank dataset. Therefore, we developed a method to measure them from standardized images of the specimens (*Methods*). The same measurements were then taken from 15 Neanderthals, 18 AMHs, and 20 *H. erectus*, which serve as reference groups (Dataset S2).

In each comparison, a test subject’s phenotype was compared to the distribution of this phenotype in the Neanderthal or AMH reference groups. The test subject’s phenotype was considered a match to the Denisovan profile if it fell on the side of the reference group’s median on which Denisovans are expected to fall. For instance, our profile suggested that Denisovans had a longer face compared to AMHs. We therefore compared facial height in each test subject against the distribution of facial heights in AMHs. If a test subject’s facial height fell above the median of facial heights in AMHs, it was considered a match (Fig. 3*C* and *SI Appendix*, Fig. S1). This method is similar in spirit to the notion of “ensemble learning” in machine learning, where a decision is achieved using a combination of “base learners” or “weak learners” (61). Here, the observation of whether a single measurement is above or below the median is a weak learner, but the strength of the final conclusions comes from combining many such weak learners.

### 2.3. Estimating the Match Between Test Subjects and the Denisovan Morphological Profile.

We estimate the degree of similarity of a specimen to the reconstructed Denisovan profile using a metric we refer to as the phenotypic distance (see full description in *Methods*). A phenotypic distance is separately computed for each phenotypic comparison to a reference group, and it is based on the quantile of the phenotype in the test subject compared to its distribution in the reference group. The phenotypic distance is scaled to (−1,1), with positive values reflecting agreement with the predicted Denisovan profile, and negative values reflecting disagreement (*SI Appendix*, Fig. S4). It is important to note that a higher positive value does not necessarily mean a better fit to the Denisovan profile, as the directional predictions are qualitative and not quantitative, and therefore, the expected phenotypic distance of Denisovan phenotypes from the reference groups is unknown. However, higher positive phenotypic distances do indicate increased confidence in the observed divergence between the test subject and the reference group.

Because measurements that match the Denisovan profile yield positive phenotypic distances, specimens resembling the Denisovan profile exhibit a distribution of phenotypic distances that is skewed toward positive values (e.g., *SI Appendix*, Fig. S4*A*). Conversely, specimens lacking resemblance to the Denisovan profile tend to display values that are either evenly dispersed around zero or skewed toward negative values (e.g., *SI Appendix*, Fig. S4*E*). As a control, we computed phenotypic distances for each specimen in the reference groups by treating it as a test subject, i.e., excluding it from its reference group and testing it against the reference groups.

We use the fact that positive phenotypic distances represent a match to the predicted Denisovan profile to develop two scores that measure the degree of concordance between each test subject and the predicted Denisovan profile (Fig. 4). The first score is based on the proportion of matches of each specimen to the profile. For each specimen, we computed a binomial score that is based on a one-tailed binomial test, using the null hypothesis that the specimen is equally likely to be higher or lower than the median of the reference group (i.e., the probability of a positive phenotypic distance is 0.5). In this analysis, higher scores correspond to a greater bias toward positive phenotypic distances, reflecting a better match to the Denisovan profile (*Methods*).

The second score accounts for the values of the phenotypic distances by testing the extent to which the median of the phenotypic distances of each specimen deviates from zero. To this end, we defined a Wilcoxon score based on a one-tailed Wilcoxon test for each specimen, which assigns higher scores to specimens that deviate more extremely in the direction of the predicted Denisovan profile across many phenotypes (*Methods*).

In summary, the binomial score primarily focuses on the number of matches, while the Wilcoxon score also factors in the measured value of the phenotype, giving more weight to more extreme differences between the test subject and the reference groups. Because the exact extent of phenotypic change in Denisovans is unknown, it remains uncertain which score better represents the match to the Denisovan profile. Consequently, and given the fact that the two scores are correlated (Fig. 4), we utilized both to rank the specimens.

### 2.4. *Harbin, Dali, and Kabwe 1* Show High Concordance with the Denisovan Profile.

As expected, the non-Denisovan groups—AMHs, Neanderthals, and *H. erectus*—exhibited score distributions shifted toward the lower end of the scale, indicating a limited resemblance to the predicted Denisovan morphology (Fig. 4 and Dataset S3). The notably low scores of *H. erectus*, which exhibits ancestral morphology compared to both AMHs and Neanderthals, highlight that ancestral morphology alone is not sufficient in order to yield high scores. Additionally, most test subjects—particularly *Steinheim*, *Ndutu*, *Eliye Springs*, and *Narmada*—received low scores similar to those of the reference groups. These results suggest that simply having a distinct or ancestral morphology relative to AMHs and Neanderthals does not guarantee high scores. Finally, in line with the closer phylogenetic relationship and greater predicted morphological similarity between Neanderthals and Denisovans (41), Neanderthals generally scored higher than the other reference groups.

Interestingly, three test subjects-*Harbin*, *Dali*, and *Kabwe 1*-received the highest scores among all 63 specimens examined in the analysis. In the binomial scoring, the strongest compatibility with the Denisovan profile was observed for *Harbin*. Out of 18 predictions that distinguish Denisovans from AMHs or Neanderthals, *Harbin* matched 16 (*SI Appendix*, Fig. S1). This represents a match rate of 88.9% (binomial score = 3.44; see x-axis in Fig. 4). In the Wilcoxon scoring, the highest compatibility with the Denisovan profile was observed for *Kabwe 1*, *Dali*, and *Harbin* (see y-axis in Fig. 4). Overall, these three specimens ranked consistently high across both scoring methods.

If the predicted Denisovan profile holds true information about Denisovan morphology, and if a test subject indeed has Denisovan-like morphology, this should be reflected by its binomial and Wilcoxon scores being significantly higher than expected by chance. We implemented a permutation test in order to *i*) validate that the measurements we used to compare test subjects to the predicted Denisovan profile are more informative than randomly chosen measurements, *ii*) control for potential biases, such as overall cranial size, correlations between phenotypes, and the number of available comparisons, and *iii*) estimate the significance of individual test subject scores. Importantly, this test obviates standard normalization techniques that rely on summary statistics of size [e.g., division by the cube root of the cranial capacity (12)], which may generate false negatives in cases of phenotypes changing in all three dimensions (*Methods*). Nevertheless, we repeated this analysis using normalized measurements and received similar results (Dataset S4).

In each permutation, we replaced phenotypes from the Denisovan profile with random ones from the Morphobank dataset (12), while maintaining the directional correlations between phenotypes. Then, we tested the match of each specimen to the randomly permuted profile. We repeated this 10,000 times for each specimen and assigned a *P*-value by computing the proportion of permutations where the match to the permuted profile exceeded that of the match to the true profile. Finally, we adjusted the *P*-values for multiple comparisons (*Methods*).

We found that most test subjects did not show a significant match to the Denisovan profile, suggesting that their morphology is no more similar to the predicted Denisovan morphology than expected by chance. However, three test subjects showed significant resemblance to the Denisovan profile: *Dali* (adj. P=0.0231), *Harbin* (adj. P=0.0264), *Kabwe 1* (P=0.0264) (Dataset S3). Similarly significant outcomes were obtained using normalized values (Dataset S4). Based on these findings, we propose that *Harbin*, *Dali*, and *Kabwe 1* present unique and significant Denisovan-like morphology. The observation that *Harbin*, molecularly confirmed as a Denisovan, significantly matches our predicted Denisovan profile (*SI Appendix*, Fig. S1) further strengthens the affinity of the other two top candidates to the Denisovan lineage.

### 2.5. Distinguishing Between Denisovan-Like and Neanderthal-Like Morphology.

The evolutionary distance between Denisovans and Neanderthals is shorter than that between Denisovans and AMHs (1), making it more challenging to distinguish between these sister groups. Indeed, the majority of predictions in the Denisovan profile (12 out of the 18) distinguish between AMHs and both Denisovans and Neanderthals (Fig. 2), while only six distinguish between Denisovans and Neanderthals. As a result, some of the high scores we observed may have been driven by matches to phenotypes placing a specimen within the Neanderthal–Denisovan clade, rather than specifically closer to Denisovans more than to Neanderthals (1).

To the best of our knowledge, none of the three top-scoring specimens are currently considered to be Neanderthals. However, they may nevertheless represent members of a yet-unknown lineage in the Neanderthal–Denisovan clade, or be close to the root of the Neanderthal–Denisovan clade. To address this possibility, we conducted an additional analysis focusing exclusively on the six predicted morphological differences separating Neanderthals and Denisovans (Dataset S2 and Fig. 2). To further refine the differentiation between Denisovans and Neanderthals, we added to these six predictions the hallmark Denisovan phenotype of *larger molar crown size*, which has been previously demonstrated to differ between Denisovans and Neanderthals (2, 5, 6, 54, 55), and was also used to infer Denisovan affinity in previous studies (16).

**Fig. 5.**
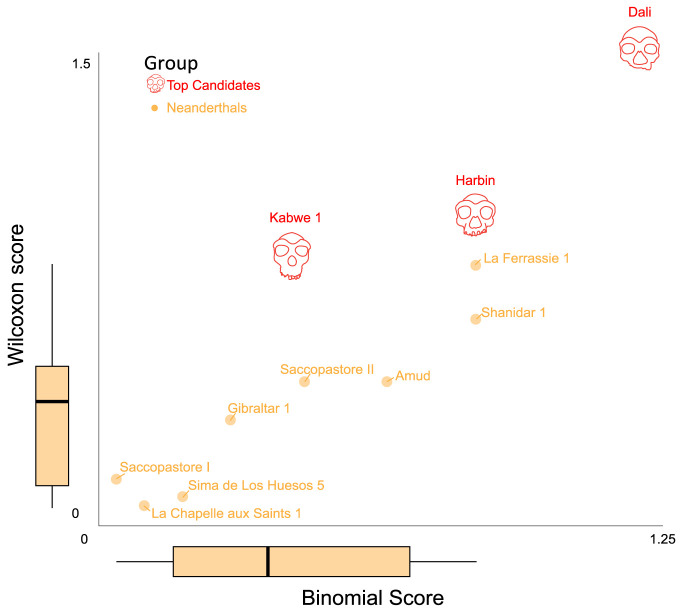
Match scores for phenotypes predicted to distinguish between Denisovans and Neanderthals. The analysis was carried out for specimens with high affinity to the Neanderthal–Denisovan clade (i.e., Neanderthals and high-scoring test subjects).

When considering both the binomial and Wilcoxon scores, *Kabwe 1* falls within the distribution of scores observed in Neanderthals (86th percentile of the Neanderthal distribution of the combined Wilcoxon and binomial scores, Fig. 5). Thus, even though *Kabwe 1* received high scores in the overall analysis, which suggested close affinity to the Neanderthal–Denisovan clade, our results do not place it closer to the Denisovan lineage than to the Neanderthal lineage. Indeed, its older dating may suggest that it is close to the root of the Neanderthal–Denisovan split (34). In contrast, *Harbin*, and to an even greater extent, *Dali*, received high scores, exceeding all Neanderthals. These results are robust to the exclusion of the molar crown size phenotype (*Methods*) and further support the affinity of *Harbin* and *Dali* to the Denisovan lineage.

### 2.6. High-Scoring Specimens Exhibit Morphological Similarity.

To explore potential morphological affinities between test subjects and examine whether distinct clusters emerge, we carried out a principal component analysis (PCA). To this end, we used all available continuous cranial measurements in the Morphobank dataset (12), with a maximum of 20% missing data points. To this, we added the four measurements generated for this study (glabellar curvature, calvarial curvature, and forehead height), leaving us with a total of 52 measurements (Dataset S5). To avoid overimputation, we also filtered out specimens with more than 15% missing measurements, leaving us with a set of 49 specimens in the final PCA. The remaining missing data were imputed using the Singular Value Decomposition (SVD) method (56). This analysis also allowed us to study *Xuchang 1* and *Narmada*, which did not have sufficient data to be compared against the Denisovan profile, but could be compared against other specimens using the 52 aforementioned measurements.

**Fig. 6.**
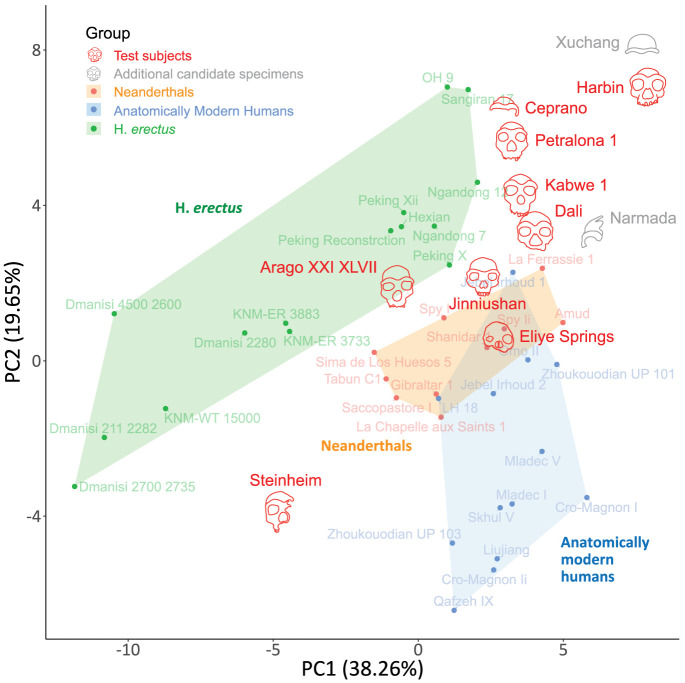
Principal component analysis (PCA) of the specimens included in the study, based on 52 available cranial measurements. In red are our test subjects. Crania in gray represent specimens with fewer than five testable phenotypes or those belonging to subadults and were therefore not tested against the Denisovan profile. The polygons depict the convex hull of the three reference groups. High-scoring test subjects cluster together, suggesting cranial morphological similarity between them.

The PCA exhibited a high level of separation between the reference groups *H. erectus*, Neanderthals, and AMHs (particularly between *H. erectus* and the other groups, Fig. 6). This suggests that these cranial measurements are able to capture the distinct evolutionary histories of these human lineages. Next, we projected the test subjects onto the PCA plane (Fig. 6). Notably, several of them were placed close to one another and separated from the reference groups. These include *Harbin*, *Xuchang 1*, *Ceprano*, *Petralona 1*, *Kabwe 1*, *Dali*, and *Narmada*. *Jinniushan* is positioned close to Dali, between the Neanderthal and *H. erectus* populations. Other test subjects were positioned either within the other populations (e.g., *Eliye Springs*), or far from any other population (*Steinheim*). The top-right cluster includes specimens that display a high concordance with the Denisovan profile, and particularly the top three *Harbin*, *Dali*, and *Kabwe 1*. This suggests that specimens resembling the Denisovan profile form a cluster of morphologically similar specimens not only with respect to their Denisovan-like phenotypes but also to the rest of their cranial morphology. Furthermore, we have carried out a second PCA, using solely nonmetric measurements, which exhibited similar clustering patterns and morphological relations between specimens (*SI Appendix*, Fig. S3). This provides further credence to their grouping in the same cluster, regardless of the overall cranial size. Interestingly, *Xuchang 1*, whose high similarity to the Denisovan profile was previously noted (41), but did not have enough measurements to be systematically tested in the current work, clusters very close to *Harbin*.

## 3. Discussion

Identifying Denisovans in the fossil record is a key step in understanding their evolution. However, this is a highly challenging task, as attested by the scarcity of confirmed Denisovan specimens. Here, we proposed that reconstructing Denisovan morphology using gene activity patterns provides a promising way to link genetics and anatomy, and opens the window to systematically scanning debated specimens and quantifying their degree of resemblance to the predicted Denisovan anatomy.

We highlighted three specimens that show a particularly high resemblance to the predicted Denisovan profile (41): *Dali*, *Kabwe 1*, and *Harbin*. Reassuringly, the latter has been recently confirmed molecularly as a Denisovan (14, 15). *Dali* has previously been suggested to possibly share links with Denisovans through circumstantial evidence (e.g., their temporal and geographical context) (1, 34). Here, we provide the first comprehensive genetics-based evidence of *Dali*’s potential link to the Neanderthal–Denisovan clade.

Some specimens, such as *Xuchang 1*, lacked sufficient measurements for inclusion in our analyses. However, this does not imply that these specimens are unrelated to Denisovans; rather, it means that our method cannot currently provide a conclusive assessment of their affinity to the Denisovan lineage. Notably, several lines of evidence support a possible Denisovan affinity for *Xuchang 1*: *i*) All four of its available predictions align with the Denisovan profile, including biparietal width–a trait we predicted to be larger in Denisovans than in both AMHs and Neanderthals. Interestingly, *Xuchang 1* has the widest biparietal width in the examined dataset (12), and the largest maximum cranial breadth in the Late Pleistocene fossil record (27), *ii*) in the PCA, *Xuchang 1* clusters most closely with *Harbin* and far from the other groups (Fig. 6), *iii*) its geographical location falls within the inferred habitat of Denisovans. It remains to be seen whether its identity can be further elucidated in the future.

Several test subjects, most notably *Kabwe 1*, exhibit unexpectedly high concordance with the Denisovan profile, despite being unlikely to be Denisovans. Although discovered in Africa, *Kabwe 1* not only aligns well with the Denisovan profile (Fig. 4) but also clusters closely in the PCA with *Dali*, a potential Denisovan (Fig. 6 and *SI Appendix*, Fig. S3). Although our current knowledge of Denisovan habitat is lacking, it is unlikely that they reached Southern Africa. Hence, *Kabwe 1* is unlikely to be directly positioned on the Denisovan lineage. A more plausible explanation is that the resemblance of some of these specimens to Denisovans reflects a proximal phylogenetic affinity with the Neanderthal–Denisovan clade, perhaps close to its root. Indeed, we found that *Kabwe 1* performed lower than *Harbin* and *Dali* when considering only Denisovan-derived phenotypes (Fig. 5). Furthermore, the phylogenetic position of *Kabwe 1* was previously suggested to be close to the split between modern humans and the Neanderthal–Denisovan clade (34). Its relatively old dating further supports a phylogenetic position close to the last common ancestor of Neanderthals and Denisovans. Overall, specimens that are phylogenetically closer to the Neanderthal–Denisovan last common ancestor than to Neanderthals are expected to exhibit a greater similarity to the Denisovan profile than Neanderthals do.

Almost all AMH and *H. erectus* specimens in our analysis received relatively low scores (Dataset S3), indicating that our method is not universally permissive, and that lineages that are relatively far from Denisovans do not tend to align with the profile. Neanderthals, who are closer to Denisovans and are thus expected to share many phenotypes with them, tend to show higher scores. Still, none of the Neanderthals received scores comparable to the top-scoring test subjects (*Harbin*, *Dali*, and *Kabwe 1*), even the two highest Neanderthals (*La Ferrassie* and *Shanidar 1*) (Figs. 4 and 5). Interestingly, two *H. erectus* (*Sangiran 17* and *Hexian*) and one AMH individual (*Irhoud 1*) also received relatively high scores (Fig. 4 and Dataset S3). While some outliers are expected statistically, as well as due to the imperfect precision of the profile, it is nevertheless interesting to note that *i*) the two *H. erectus* specimens are East Asian, and, *ii*) the AMH individual is the oldest AMH specimen (62), thus closest to the common ancestor of AMHs, Neanderthals and Denisovans, and it previously reported to be similar to *Dali* (63). Overall, the rare occurrence of false positives, the high precision of the anatomical profiling (>85%)(41), and the observation that 25 out of 28 (89%) predictions have now been validated by Denisovan specimens (11, 12, 57) support the robustness of this method in detecting Denisovan-like morphology.

The Denisovan morphological reconstruction (41) is based on a comparative analysis between AMHs, Neanderthals, and a single Denisovan specimen. We acknowledge that the morphology of a single individual cannot encompass the spatiotemporal morphological variation of an entire population. This is especially relevant in the case of Denisovans, who had a complex population structure, with deeply divergent lineages that separated as early as 350 Kya (64). This divergence, along with the wide spatial distribution of Denisovans in Asia, likely resulted in morphological variability within Denisovans (1, 30). As a result, our reconstruction approach is likely to miss some nonfixed derived Denisovan phenotypes. However, most predictions in the original reconstruction, as well as in this work, stem from either AMH-derived or archaic-derived changes, suggesting that they are synapomorphic in Denisovans. In fact, only three measurements used in this work are strictly based on Denisovan-derived phenotypes (palate length, glenoid fossa area, and biparietal breadth). Therefore, the morphological profile tested in this work likely consists of phenotypes that are shared by most Denisovans.

Relatively few derived phenotypes separate Denisovans from Neanderthals (three Denisovan-derived and three Neanderthal-derived). This could lead to less power in separating the two groups using our method. However, we have shown that the top-scoring test subjects are distinct from Neanderthals in several ways: *i*) No Neanderthal receives scores higher than the top-scoring test subjects; *ii*) we were able to differentiate groups within the Neanderthal–Denisovan clade using derived phenotypes and predictions within the Neanderthal–Denisovan clade; *iii*) last, we showed that the overall morphology of top-scoring test subjects tends to be similar, and they clustered separately from Neanderthals (Fig. 6).

Our work showcases the potential of gene regulatory information as a powerful predictive tool for inferring complex phenotypes. Using genetic phenotyping, we were able to link fossils lacking genetic information to a genetically defined lineage that lacks fossils. By demonstrating the robustness of this approach, we envision that similar methodologies will become instrumental in inferring the phenotypes of other extinct Hominin groups, and assist in future classification of the fossil record, once new methylation maps are available. Similar methods could also be applicable to other extant pairs of closely related primate species (42). As our understanding of gene regulation deepens, and as insights into ancient gene regulation in nonskeletal tissues emerge (36, 38–40, 65, 66), we anticipate that these techniques will expand beyond the skeletal system to include tissues that are not accessible through traditional methods.

## Supplementary Material

Appendix 01 (PDF)

Dataset S01 (XLSX)

Dataset S02 (XLSX)

Dataset S03 (XLSX)

Dataset S04 (XLSX)

Dataset S05 (XLSX)

Dataset S06 (XLSX)

## Data Availability

The code used to measure phenotypic values directly from images is available at https://github.com/gadiherz/CraniaAnalysis (67). The code used for the analysis is available at https://github.com/gokhmanlab/DenisovanCraniaProject (68). All other data are included in the manuscript and/or supporting information. Previously published data were used for this work (Morphobank).
