# Supplementary material for: Candidate Denisovan fossils identified through gene regulatory phenotyping
Source: Proc Natl Acad Sci U S A. 2025 Aug 26;122(35):e2513968122. doi: 10.1073/pnas.2513968122 (PMC12415249; doi:10.1073/pnas.2513968122)
Supplement: Supplementary file 1 — Appendix 01 (PDF) [file pnas.2513968122.sapp.pdf]

Supporting Information

1 Cranial Measurements

1.1 Palate breadth

The Denisovan palate was predicted to be wider than that of AMHs based on the *Narrow palate* phenotype [HP:0000189]. This phenotype is described as decreased palatal width. Here, we measured it using maxilloalveolar breadth (MAB), defined as the greatest breadth across the alveolar border, perpendicular to the medial plane [1].

1.2 Facial breadth

The Denisovan face was predicted to be wider than that of AMHs and narrower than that of Neanderthals based on the *Small face* phenotype [HP:0000274] and the *Narrow face* phenotype [HP:0000275], which is hierarchically embedded within it. Here, we measured these phenotypes using bizygomatic breadth (ZYB, zy-zy), similarly to previous measurements of the breadth of the upper face [2].

1.3 Facial height

The Denisovan face was predicted to be longer than that of AMHs based on the *Short face* phenotype [HP:0011219], which is hierarchically embedded within the *Small face* phenotype [HP:0000274]. This phenotype can be measured as the vertical distance between the nasion to the gnathion (the inferior border of the mandible) [2]. However, as the test subjects lacked a mandible, this measurement was unavailable. Instead, we used upper facial height (NPH),

defined as the vertical distance from the nasion to the prosthion.

1.4 Biparietal breadth

The Denisovan parietal bones were predicted to be more laterally expanded than those of both AMHs and Neanderthals based on the *biparietal narrowing* phenotype [HP:0004422]. We measured this using maximum biparietal breadth [1].

1.5 Cranial base area

Denisovans were predicted to have a larger cranial base area than that of AMHs based on the *decreased cranial base ossification* phenotype [HP:0005451]. The cranial base is the supporting bony structure behind the midface (e.g., [3]). To estimate cranial base size, we approximated it by the area of an ellipse with one axis measured by the basion-nasion length (BNL) and the other by the biauricular breadth (AUB). Therefore, $A_{\text{cranial base}} = \pi \times \text{AUB} \times \text{BNL}$, with both measurements taken from [1] (Dataset S6).

1.6 Dental arch length

Denisovans are predicted to have less crowded dental arches than those of both AMHs and Neanderthals, based on the *dental crowding* phenotype [HP:0000678], described as an inadequate arch length for tooth size usually present before M^3 eruption. Reduced dental crowding can be a result of three causes: (i) increased length of the dental arch; (ii) reduced number of teeth; or (iii) smaller teeth. The dental formula of hominins is highly consistent [4], making the second cause unlikely. The third cause is also unlikely, as the confirmed Denisovan teeth are much larger than in other *Homo* lineages [5]. We therefore concluded that the

Denisovan dental arch was likely longer than than that of AMHs and Neanderthals. To test dental arch length in the maxilla, we used the maxilloalveolar length, defined as the greatest length of the alveolar process of maxilla [1].

1.7 Facial protrusion

Denisovans are predicted to have more protruding faces than those of AMHs, but less than those of Neanderthals, based on the *flat face* phenotype [HP:0012368]. The convexity (or concavity) of the face is examined in lateral view [2]. To measure facial protrusion, we used the nasion angle (NAA) and prosthion angle (PRA). Both measurements are used as measures of prognathism [1]. Since these angles were not provided in the database, we calculated them using the law of cosines and the relevant facial measurements that were provided (NPH, BNL, BPL). The two measurements (PRA, NAA) were combined into a single value - the protrusion index, defined as the first principal component (Dataset S6). This component explained 94% of the variability in the two measurements. We chose to use PCA over linear regression, as the latter assumes univariate errors (only in measuring the dependent variable), whereas in our case both variables are associated with errors.

1.8 Glenoid fossa size

Denisovans were predicted to have a larger mandibular condyle than both AMHs and Neanderthals. The prediction is based on the *mandibular condyle hypoplasia* phenotype [HP:0007628], which reflects a general decrease in the mandibular condyle size. Most specimens included in the analysis do not have a mandible. However, the condyle forms the mandibular part of the temporomandibular joint, where it articulates with the *glenoid fossa* of the temporal bone [6]. As with all synovial joints, these bony surfaces are separated by several thin layers of soft tissues, with the articular disc being the main component. Nevertheless,

it has been shown that glenoid fossa morphogenesis is dependent on signals from the developing mandibular condyle [7], likely increases in size to accommodate a larger condyle [8], and was shown to be a good proxy for condylar head size [9].

We identified three measurements related to the dimensions of the glenoid fossa: (1) ectoglenoid-entoglenoid length (ect-ent), (2) postglenoid-ectoglenoid length (pos-ect) and (3) postglenoid-entoglenoid length (pos-ent) [1]. We first used these values to calculate the semi-perimeter of the triangle:

$$s = \frac{(ect - ent) + (pos - ect) + (pos - ent)}{2}$$

and then used Heron's formula to calculate the area of the triangle between the landmarks ectoglenoid, entoglenoid and postglenoid:

$$A = \sqrt{s \cdot (s - (ect - ent)) \cdot (s - (pos - ect)) \cdot (s - (pos - ent))}$$

(*SI Appendix*, Fig. S5). We use the area of the triangle as a proxy for the glenoid fossa size (Dataset S6).

2 Measurements Based on Cranial Images

Several measurements could not be directly matched to a suitable continuous variable from the available dataset, which offered only a discrete measurement [1]. In this work, we opted to use continuous measurements for two key reasons: (1) our analysis relies on determining a central tendency, which is best represented on a continuous scale, and (2) the discrete classification of continuous phenotypes is often subjective. Therefore, we used the available images from Ni *et al.* [1] to generate matching continuous measurements. The generated values for all these measurements can be found in Dataset S6.

The procedures for calculating calvarial flatness, forehead height, malar flattening and glabellar protrusion were applied to images of the crania [1], assumed to be consistently placed in a standard anatomical position following the Frankfurt planes. Images were provided in PNG or JPEG format. All images underwent a vectorization procedure, converting each non-white pixel into (x, y) coordinates. Pixels were considered white if the magnitude of the difference vector between their RGB values and standard white $(255, 255, 255)$ was smaller than 20. The skull boundary was extracted using the Matlab alpha shape boundary algorithm with a 0.9 shrink factor. This provided an ordered list of points $(x_i, y_i), i = 1 \dots N$ on the closed curve forming the outer boundary of the cranium.

Next, we represent each closed curve as a series of Fourier coefficients [10]. For this, we think of the coordinates of each vertex in each of the two dimensions as a unique function of the arc length denoted as s, with $0 \leq s \leq L$, where $L = \sum_{i=1}^{N-1} \sqrt{(x_{i+1} - x_i)^2 + (y_{i+1} - y_i)^2}$. As N must be an even number for the subsequent procedure, in case it is odd, one vertex is removed. For convenience, the vertices are shifted by interpolation along the curve so that the distance between each two vertices is constant. A discrete Fourier series is then fitted onto the points $x(s), y(s) = \sum_n (A_n^{x,y} \sin\left(\frac{2\pi n}{L} s\right) + B_n^{x,y} \cos\left(\frac{2\pi n}{L} s\right))$, with $n = 0, \dots, \frac{N}{2}$ for each dimension (x, y). The fitting is performed by solving a system of linear equations through matrix inversion. This provides a list of Fourier coefficients $(A_n$,$B_n)$ for each of the two dimensions, which allows to express the coordinates of the curve for each arc-length value.

The maximal resolution in which the curve can be expressed equals the shortest wavelength of the Fourier series $\frac{2L}{N}$. However, for some applications, for example, finding prominent low-level features such as locations of curvature peaks, it is useful to reduce the curve's resolution. This is achieved by introducing a smoothing factor [10] that provides a weight to each term in the series $x(s), y(s) = \sum_n w_n \left(A_n^{x,y} \sin\left(\frac{2\pi n}{L} s\right) + B_n^{x,y} \cos\left(\frac{2\pi n}{L} s\right)\right)$ where $w_n =$

$1/\left(1+e^{\frac{n-N_{\text{eff}}}{\Delta}}\right)$. N_{eff} is a constant chosen to reflect the number of terms in the series to be considered, as $w_n \approx 1$ for $n < N_{\text{eff}}$ and $w_n \to 0$ when $n > N_{\text{eff}}$. Δ reflects an interval over which this transition gradually occurs.

The Fourier series, which enables the expression of the (x, y) coordinates as a function of arc-length s can be differentiated [10]. Its first derivative is used to express the tangent angle as a function of the arc-length $T(s) = \tan^{-1}\frac{y'(s)}{x'(s)}$. Its second derivative is used to express the curvature as a function of the arc-length $C(s) = -\frac{y'x''-x'y''}{x'^2+y'^2}$.

2.1 Calvarial curvature

Denisovans were predicted to have a calvarium (top of the cranium) that was flatter in lateral view than that of AMHs, based on the *oxycephaly* phenotype [HP:0000263]. Here, we measured calvarial curvature using a lateral image of the cranium. Notably, the lateral images used here were previously used for a discrete evaluation of a similar phenotype by Ni *et al.* [1], indicating their fit for this sort of analysis. The anterior boundary of the cranial top was set to be the supraorbital sulcus, defined as a local negative minimum of the curvature function and denoted s_0. The posterior boundary point, denoted s_r was set to be the mirror-reflected projection of the supraorbital sulcus on the posterior part of the cranium. This point could have been identified automatically from the curvature function. However, many crania were fragmented in different ways, which prevented an anatomically consistent choice. To avoid such inconsistencies, a semi-automatic approach was used in which we selected the correct local minima corresponding to supraorbital sulcus out of several potential points using a graphical user interface. The potential points are those for which there is a local negative minimum in the curvature function and whose x coordinate is positive, as the supraorbital sulcus is concave and anteriorly positioned. The identification of the local minima is done with $N_{eff} = 45$ and

a buffer of 3% of the length of the clavarium around the minima, allowing the accurate identification of low-level features while filtering out points generated due to noise.

Once s_0, is selected, the arc length values corresponding to the superior cranial curve segment are plugged into the function to calculate 300 equidistant points on the curve under low smoothing with N_{eff} equals to 80% of the number of terms in the Fourier series. These points are then scaled to centroid size $CS = \sqrt{\sum_{i=1}^{n}\left((x_i - \bar{x})^2 + (y_i - \bar{y})^2\right)}$ corresponding to the mean of all points on the curve. The scaled curve segment points are then fitted with the function $x(s), y(s) = A^{(x,y)} \cos\left(\frac{s}{L}\right) + B^{(x,y)} \sin\left(\frac{s}{L}\right) + C^{(x,y)} \left(\cos\left(\frac{s}{L}\right)\right)^3 + D^{(x,y)} \left(\sin\left(\frac{s}{L}\right)\right)^3$ by solving an overdetermined system of linear equations. This function was chosen due to the balance it provides between compactness and error rate, allowing the expression of the general morphological aspect of the calvarium while smoothing local irregularities that may result from post-depositional damage. Similarly to the Fourier series, this function too is differentiated twice to calculate the tangent and curvature as a function of arc length.

The flatness value CF was calculated as the integral of the curvature's absolute value over the curve segment representing the calvarium. This provides cranial flatness value of $CF = \int_{s_0}^{s_r} |C(s)| ds$, such that lower values reflect a flatter segment (*SI Appendix*, Fig. S 6 *A* and *B*).

Whenever possible, the flatness value CF was calculated for both lateral right and lateral left images. This measurement could not be computed for crania in which neither of the lateral images was intact (namely, the calvarial outline is complete and continuous). In crania where only one side was intact, only that side was used. In crania where both sides were intact, their average was used. We also compared the values produced from each side to examine the effectiveness of this method (*SI Appendix*, Fig. S7); The error was calculated by Left-Right. One specimen (Peking 12) with outlying error values (Error $>$ 2 standard deviations) was removed from further analysis. Overall, data that was

available for both sides showed high Pearson correlation between them ($r = 0.93$, $p = 2.2 \cdot 10^{-16}$) (*SI Appendix*, Fig. S7), indicating high consistency.

Our metric for calvarial curvature serves as a quantitative refinement of a similar discrete estimation in Ni *et al.* [1], who looked at the convexity along the sagittal profile of the frontal bone between the supratoral sulcus and the bregma [Discrete phenomic character #419] [1]. Then, they classified the lateral images as either *flat*, *slightly convex* and *strongly convex*. We showed that our continuous measurement is well compatible with the discrete categorization of Ni *et al.* [1] using the Kruskal-Wallis rank sum test (*SI Appendix*, Fig. S8). This test was conducted in R using the Kruskal test function. The results revealed a statistically significant difference between the three groups ($P < 8.98 \times 10^{-7}$). Post-hoc analysis (Dunn test, using dunnTest() function [11] in the FSA package [12] in R) revealed that the means of all groups significantly differed from one another (*strongly convex-flat* adj. $P = 4.19 \times 10^{-7}$,*slightly convex-flat* adj. $P = 1.41 \times 10^{-3}$, *strongly convex-slightly convex* adj. $P = 9.87 \times 10^{-3}$).

2.2 Forehead height

Denisovans were predicted to have a lower forehead than that of AMHs based on the *high forehead* phenotype [HP:0000348]. Similarly to the calculation of calvarial curvature, we used lateral images of the crania. A series of 300 equidistant points on the calvarium curve were calculated using the Fourier coefficients under a smoothing of $N_{eff} = 14$. Then, the tangent function was used to identify the cranial vertex s_v, defined as $\arg\min_{s \in [s_0, s_r]} |T(s)|$. It was also used to locate the forehead point s_{fr}, which was arbitrarily defined as $\arg\min_{s \in [s_0, s_v]} |T(s) - \frac{1}{4}\pi|$. This allowed the calculation of the forehead height $FH = y(s_{fr}) - y(s_0)$.

Similarly to the values of calvarial curvature, we evaluated our measurement of forehead height by comparing the right and left lateral images (*SI Appendix*,

Fig. S9). Two specimens with outlying differences between the two sides were removed (Ngandong 7 and Steinheim). Then, the estimates drawn from the two sides of each individual showed high Pearson correlation ($r = 0.82$, $P = 2.35 \times 10^{-12}$), indicating high consistency of the method.

2.3 Glabellar curvature

Denisovans (as well as Neanderthals) were predicted to have a retracted glabellar region compared to that of AMHs. This phenotype was originally omitted from the reconstruction [13], due to its interpretation as the absolute protrusion of the glabella, together with the supraorbital torus. Since Neanderthals are known to have a more projecting glabella than that of AMHs [14, 15], this prediction was first evaluated to be incorrect. However, after re-evaluation, we concluded that *Glabellar protrusion* in HPO refers to the protrusion of the glabellar region relatively to the rest of the supraorbital torus, and not in absolute terms [2]. The relative position of the glabella affects the curvature of the immediate glabellar region when observed in anterior and superior views. Indeed, the Neanderthal glabella was previously described as more anteroinferiorly positioned compared to AMHs and African H. *erectus*, resulting in a more receding mid-sagittal supraorbital region [15], creating a double-arched shaped supraorbital torus in Neanderthals. Since the predicted phenotype aligns with this description, we have decided to treat this as a correct prediction, in contrast with the original analysis [13].

Here, we measured glabellar curvature using superior images of the cranium, assuming that all crania were placed in a consistent anatomical position. For this, we only used crania that had an intact supraorbital torus in the midsagittal plane. In cases where the midfacial bones were apparent in superior view, they were digitally removed. This procedure was done for the following specimens: Sima de los Huesos 5, Dmanisi 2282, Dmanisi 2700, Saccopastore 1, Shanidar 5,Bodo, ER-1805 and OH 24. The last three specimens were not included in

the final analysis as test subjects or as part of the reference groups, but were nevertheless used for the validation of our measure of glabellar curvature (see below).

To derive a measurement of glabellar curvature, the boundary of the cranium and its Fourier coefficients were extracted using the same procedure detailed above with a smoothing factor $N_{eff} = 20$. Given the cranial morphology, the boundary curve centering and the assumption of consistent rotation, the arc length value of the glabella s_{gl} was that for which $x(s_{gl}) > 0, y(s_{gl}) = 0$, or in other words the intersection of the curve and the x axis in its positive part. Thus, the glabellar region was defined as the segment of the curve spanning $s_{gl} \pm \frac{L}{25}$ (*SI Appendix*, Fig. S6 *C*). The value of the curvature function was calculated for s_{gl}, as well as for another 20 equidistant points covering this segment. The glabellar curvature is expressed as $C(s_{gl})$. In cases where there was noticeable cranial asymmetry that prevented s_{gl} from corresponding to the anatomical glabella, we selected the value of the correct point out of the 20 points for which curvature was calculated. The glabellar curvature values for all groups are presented in *SI Appendix*, Fig. S6 *C*.

Similarly to calvarial curvature, here too a similar discrete metric was used by Ni *et al.* [1], termed "glabella concavity relative to the supraorbital tori". This metric was based on the superior view images and included three categories: *deep*, *shallow*, and *absent*. Here too, we used the Kruskal-Wallis test to check for consistency between the discrete metric of Ni *et al.*[1] and our continuous measurement (S*SI Appendix*, Fig. S11). In support of this consistency, the three groups did not have identical medians ($P < 3.04 \times 10^{-7}$). Post-hoc analysis (Dunn test) revealed that the *absent* group is significantly different from the other groups (*absent*-*deep* adj. $P < 1.74 \times 10^{-6}$, *absent*-*shallow* adj. $P = 3.31 \times 10^{-4}$). However, the *shallow* and *deep* groups do not significantly differ from each other (adj. $P = 0.266$). Overall, these results serve as further support for the effectiveness of the method we developed.

The curves used to calculate calvarial curvature, forehead height, and glabellar curvature can be found in *SI Appendix*, Fig. S12. Examples of the curves used for the reference groups can be found in *SI Appendix*, Fig. S13.

2.4 Malar flattening

Denisovans were predicted to have a malar region more flattened than that of AMHs, but less flattened than that of Neanderthals, based on the *malar flattening* phenotype [HP:0000272]. This prediction was included in the original reconstruction but was merged with *hypoplasia of the midface* [HP:0011800]. In the current work, we deemed it as a sufficiently distinct phenotype based on the fact that more midfacial protrusion does not necessarily result in a more flattened malar region [16].

To compute malar flattening, we apply a similar procedure to previous subsections. Namely, the boundary curve was identified and extracted from inferior view images, and their Fourier coefficients were calculated. Similarly to the calculation of calvarial curvature, the user is prompted using a graphical interface to select the anterior and posterior points delineating the zygomatic arches. Here too, this procedure is semi-automatic such that the user selects the points out of a limited number of possibilities. These potential points correspond to local curvature minima identified using a smoothing factor of $N_{eff} = 35$, allowing an accurate selection of the correct point.

The point in which the general profile of the zygomatic arch begins to extend toward the posterior part of the cranium should have a prominent anterior and lateral position. To provide a quantitative estimation of malar flattening, this point, denoted as S_{crit} is identified using a number of distinct steps. First, the point is identified as a curvature maxima using a smoothing factor of $N_{eff} = 17$. While this is a fairly strong smoothing, in the vast majority of cases more than a single peak is identified along the arch. In such cases, the most anterior and

lateral point is selected using a cost function giving a slightly higher importance to anteriority over laterality such that $S_{crit} = \arg\max(0.6x + 0.4y)$ of all the identified curvature maxima. Once a single point is selected, and assuming it corresponds to the true critical point, its positioning is refined by finding the closest curvature peak under a reduced smoothing factor of $N_{eff} = 27$.

The user-selected anterior point and the identified critical point define a curve section corresponding to the anterior part of the zygomatic arches. To quantitatively describe malar flattening, a line is fitted to this curve using least-squares linear regression. To avoid a bias that may be caused by strong deviations from linearity around the anterior and critical points, the line is fitted only to the central 80% portion of the curve by trimming the first and last 10%.

The angle between the fitted line and the x axis is then calculated. Angles closer to $\frac{\pi}{2}$ depict high values of malar flattening, whereas angles closer to π represent lower values of malar flattening.

Malar flattening was not computed for specimens if (i) the supraorbital torus was visible in inferior view (*Peking RC*, *Oase 2*); or (ii) the malar region is too heavily damaged or completely missing on both sides (*LH 18*, *Shanidar 1*, *Ndutu*, *Spy II*). For two specimens (*Gibraltar 1* and *Steinheim*), in which only one side was sufficiently intact, the value of the damaged side was replaced with the value of the intact side.

In several cases, a mid-zygomatic breakage was incorrectly selected by the procedure as the point of maximum curvature. Specimens with such an error on both sides were removed (*Qafzeh IX* and *Cro-Magnon II*). In cases where only one side was correctly selected, the value of the incorrect side was replaced with the correct one (*Kabwe*, *Petralona 1*, *KNM-WT-15000*, *Irhoud 1*, *Dali*). Specimens with correctly placed values on both sides had a Pearson correlation of 0.58 between the two sides, which is likely partially explained by non-symmetrical post-depositional alterations (*SI Appendix*, Fig. S14). For *Harbin* we used the

image presented in the in Ni *et al.*[1] Main figure 2.F.

The two sides were combined into a single value – the flattening index (*SI Appendix*, Fig. S15), defined as their first principal component. This component explained 83.4% of the variability in the two measurements. Similarly to the measured values of facial protrusion, we chose PCA over linear regression since the latter assumes univariate errors (only in measuring the dependent variable), whereas in our case, both variables are associated with errors.

3 Bibliography

References

1. Ni X et al. Massive cranium from Harbin in northeastern China establishes a new Middle Pleistocene human lineage. The Innovation 2021 Aug; 2:100130
2. Allanson JE et al. Elements of Morphology: Standard Terminology for the Head and Face. American journal of medical genetics. Part A 2009 Jan; 149A:6–28
3. Wu XP, Xuan J, Liu Hy, Xue Mr, and Bing L. Morphological Characteristics of the Cranial Base of Early Angle's Class II Division 1 Malocclusion in Permanent Teeth. International Journal of Morphology 2017 Jun; 35:589–95
4. Das H, Motghare V, and Singh M. Human Evolution of the Teeth & Jaws: A Mouthful of History. 2021 Jun
5. Reich D et al. Genetic history of an archaic hominin group from Denisova Cave in Siberia. en. Nature 2010 Dec; 468:1053–60

6. Alomar X et al. Anatomy of the Temporomandibular Joint. Seminars in Ultrasound, CT and MRI. Temporomandibular Joint 2007 Jun; 28:170–83

7. Wang Y et al. Tissue interaction is required for glenoid fossa development during temporomandibular joint formation. en. Developmental Dynamics 2011; 240:2466–73

8. Hinton RJ and Carlson DS. Temporal changes in human temporomandibular joint size and shape. en. American Journal of Physical Anthropology 1979; 50:325–33

9. Mehta S et al. Three-dimensional Assessment of Temporomandibular Joint Volume, and Condylar and Glenoid-fossa Morphology: A Cone-beam Computed Tomography Study. Contemporary Clinical Dentistry 2023; 14:256–64

10. Saragusti I, Karasik A, Sharon I, and Smilansky U. Quantitative analysis of shape attributes based on contours and section profiles in artifact analysis. Journal of Archaeological Science 2005 Jun; 32:841–53

11. Dinno A. dunn.test: Dunn's Test of Multiple Comparisons Using Rank Sums. 2024 Apr

12. Ogle DH, Doll JC, Wheeler AP, and Dinno A. FSA: Simple Fisheries Stock Assessment Methods. 2023 Aug

13. Gokhman D et al. Reconstructing Denisovan Anatomy Using DNA Methylation Maps. Cell 2019 Sep; 179:180–192.e10

14. Clement AF, Hillson SW, and Aiello LC. Tooth wear, Neanderthal facial morphology and the anterior dental loading hypothesis. Journal of Human Evolution 2012 Mar; 62:367–76

15. Harvati K, Hublin JJ, and Gunz P. Evolution of middle-late Pleistocene human cranio-facial form: A 3-D approach. Journal of Human Evolution 2010 Nov; 59:445–64

16. Rak Y. The Neanderthal: A new look at an old face. Journal of Human Evolution 1986 Mar; 15:151–64

4 Supplementary Figures

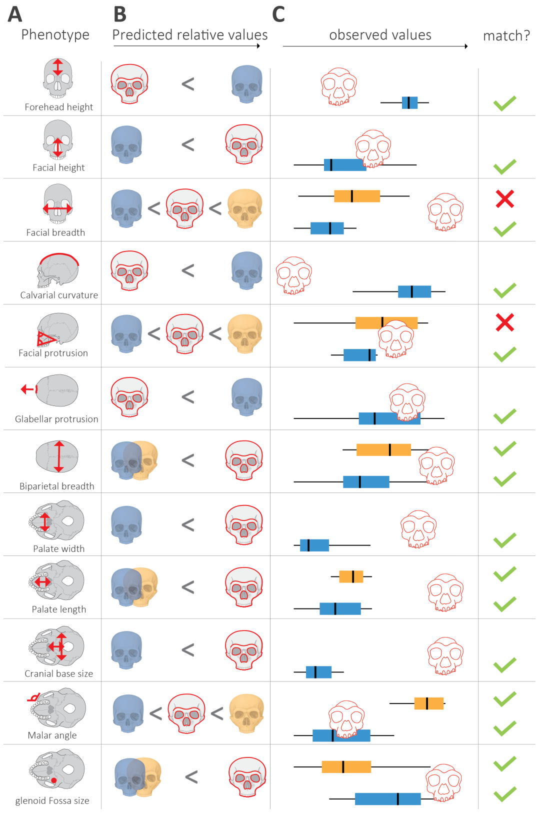

Supplementary Figure 1: Comparing the morphology of test subjects to the predicted Denisovan morphology. **A.** The twelve phenotypes used to evaluate the match of each test subject to the predicted Denisovan profile. Each phenotype includes either one or two predictions, based on whether the phenotype in the Denisovan is predicted to differ from AMHs only or from both AMHs and Neanderthals. **B.** Predicted relative value of each phenotype in Denisovans (red outline), compared to AMHs (blue) and Neanderthals (yellow). **C.** Box plots showing the distribution of each measurement in AMHs (blue) and Neanderthals (yellow). The red cranium shows the value of this measurement in a test subject, here demonstrated using *Harbin*. Check and cross marks show whether or not the *Harbin* cranium matches the predicted Denisovan position relative to the other groups.

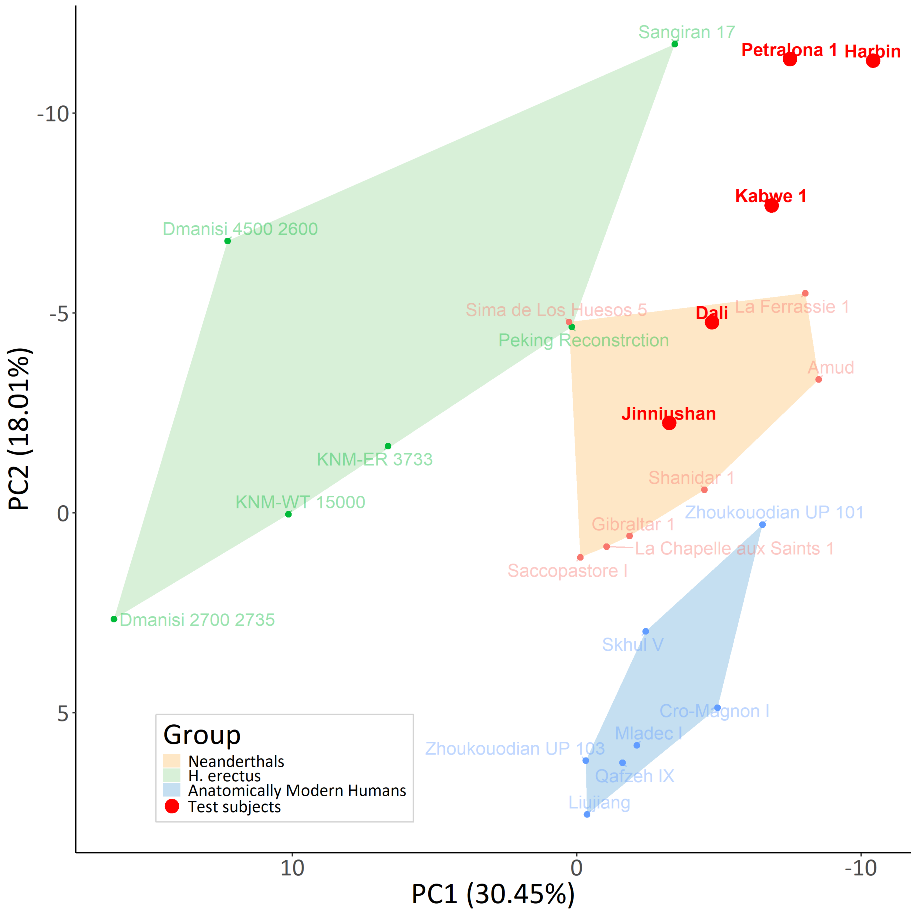

Supplementary Figure 2: PCA of the specimens included in the study, based on 141 available cranial measurements. The polygons draw the convex hull of the three reference groups. The filtering steps in this analysis are flipped compared to the main PCA; First, specimens with more than 15% missing measurements were filtered out, and only then measurements with more than 20% missing data were filtered. This order of steps enabled the inclusion of more measurements, but left out more specimens. Similarly to the main PCA, most test subjects are positioned outside the reference group clusters. However, here, Dali is positioned within the convex hull of Neanderthals.

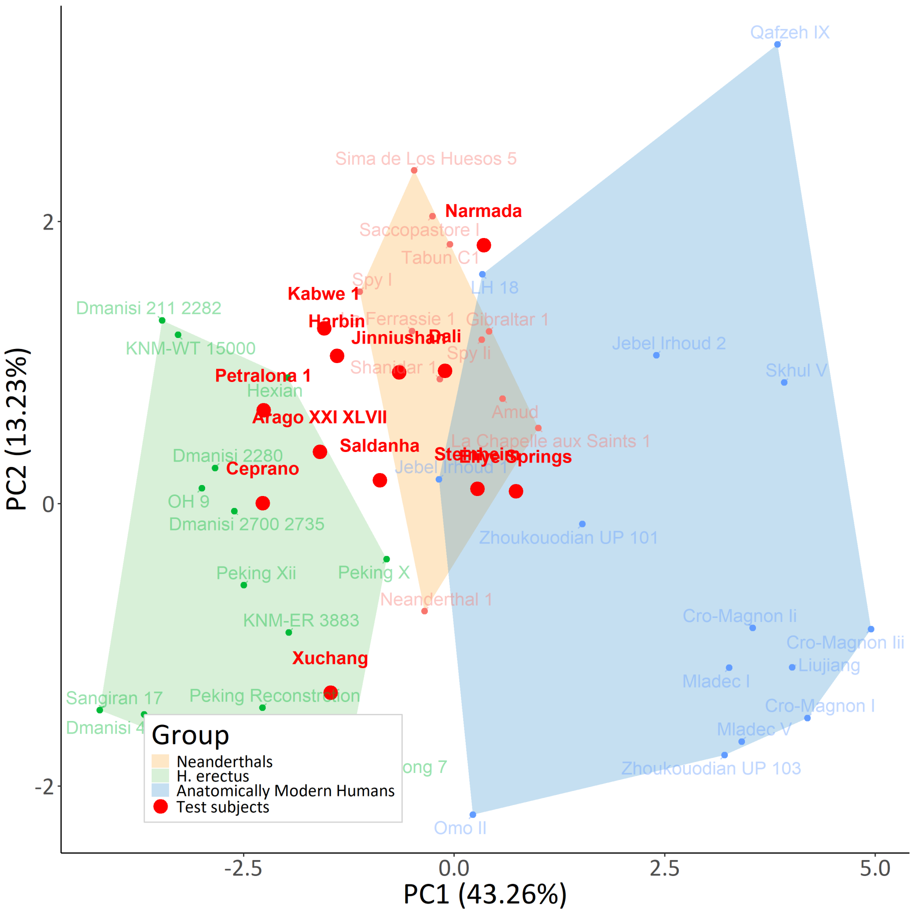

Supplementary Figure 3: PCA of the specimens included in the study, based on 15 available cranial non-metricmeasurements. The polygons draw the convex hull of the three reference groups. In this analysis, we excluded all metric measurements, leaving only angles and ratios, after applying the filtering described for the main PCA. In this PCA, the reference groups remain mostly separated. However, most test subjects in this analysis fall within the convex hulls of the reference groups.

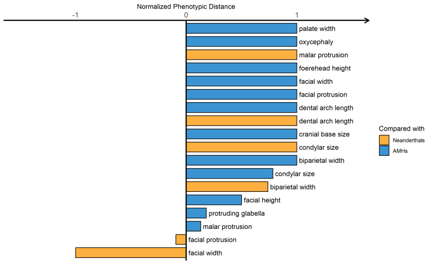

(a) *Harbin* ($N = 14$)

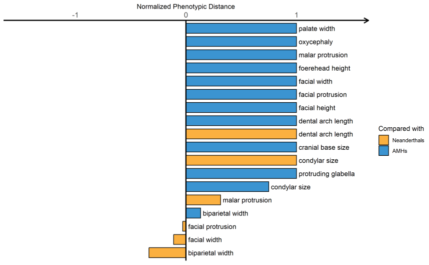

(b) *Kabwe 1*

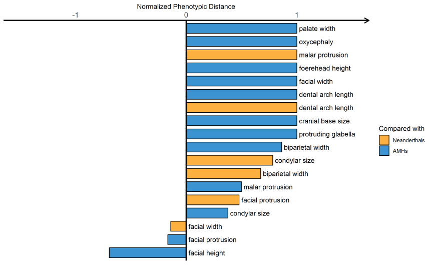

(c) *Dali*

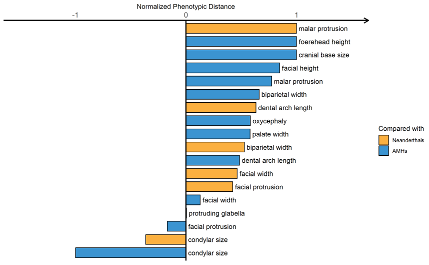

(d) *Shanidar 1*

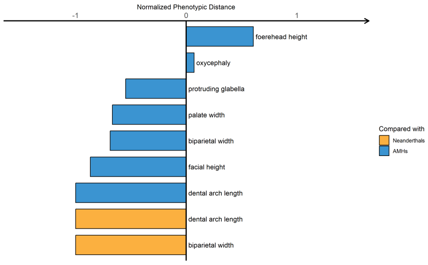

(e) *Cro-Magnon II*

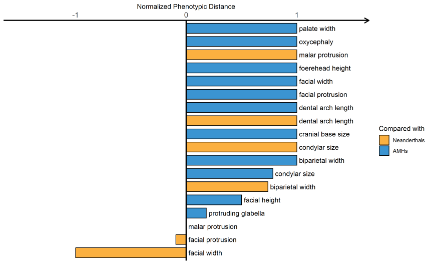

(f) *Harbin* (with alternative malar flattening value)

Supplementary Figure 4: Overall match of selected specimens to the Denisovan profile, represented as stacked histograms of phenotypic distances. Blue bars stand for measurments that are compared to AMHs, yellow bars stand for measurements that are compared to Neanderthals. (a) For *Harbin*, 12 out of 14 predictions align with the Denisovan profile. (b) For *Kabwe 1*, 11 out of 14 predictions align with the Denisovan profile. (c) For *Dali*, 11 predictions align with the Denisovan profile. (d) For *Shanidar 1*, 13 out of 14 predictions align with the Denisovan profile. (e) For *Cro-Magnon II*, 2 out of 9 predictions align with the Denisovan profile. (f) *Harbin* using the original inferior image for calculating malar flattening (see methods)

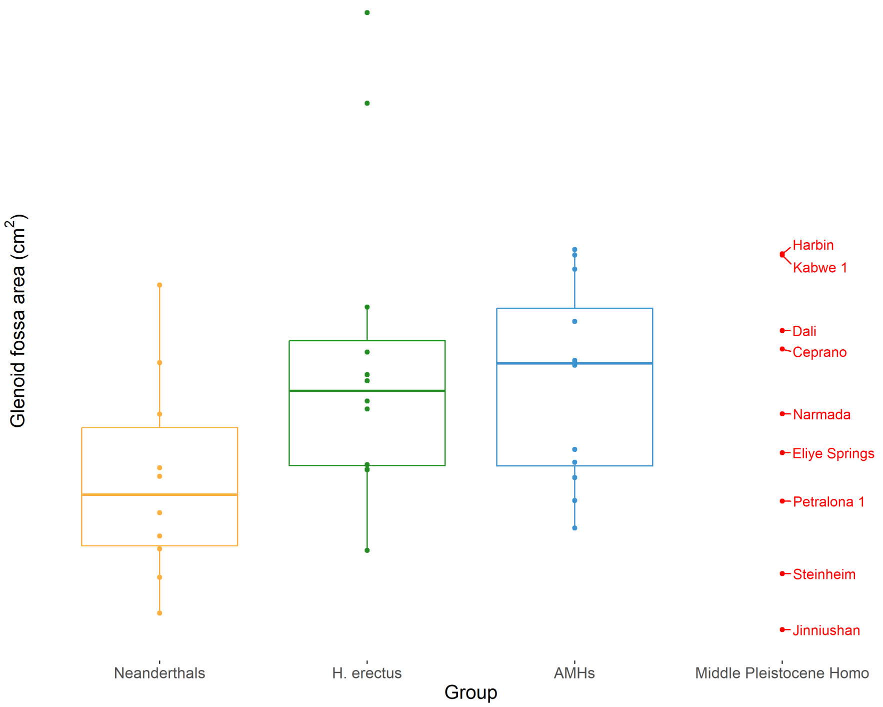

Supplementary Figure 5: Distributions of the estimated glenoid fossa area for each of the human lineages, as well as their value in the test subjects.

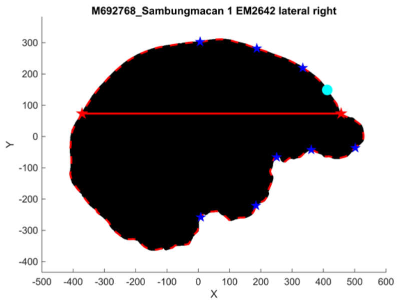

(a)

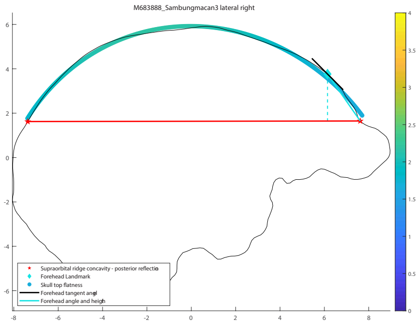

(b)

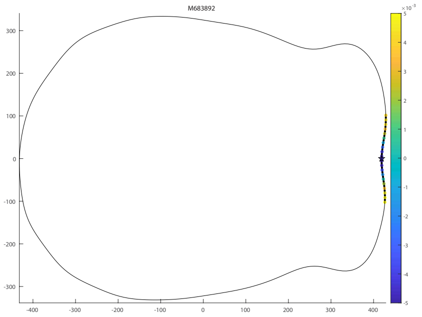

(c)

Supplementary Figure 6: Illustration of the procedure for obtaining measurements from cranial images, with specimen Sambugmacan 1 as an example. (a) Graphical user interface for selecting critical points in the lateral view. The calvarial region is defined by the outline curve above the segment connecting the critical point (right red star) to its posterior reflection (left red star). The cyan circle indicates the position of the forehead point. (b) Cranial top flatness values displayed using a color gradient, with the forehead height marked by a dashed line. (c) Curvature of the glabellar region in the superior view, with the glabella position indicated by a star.

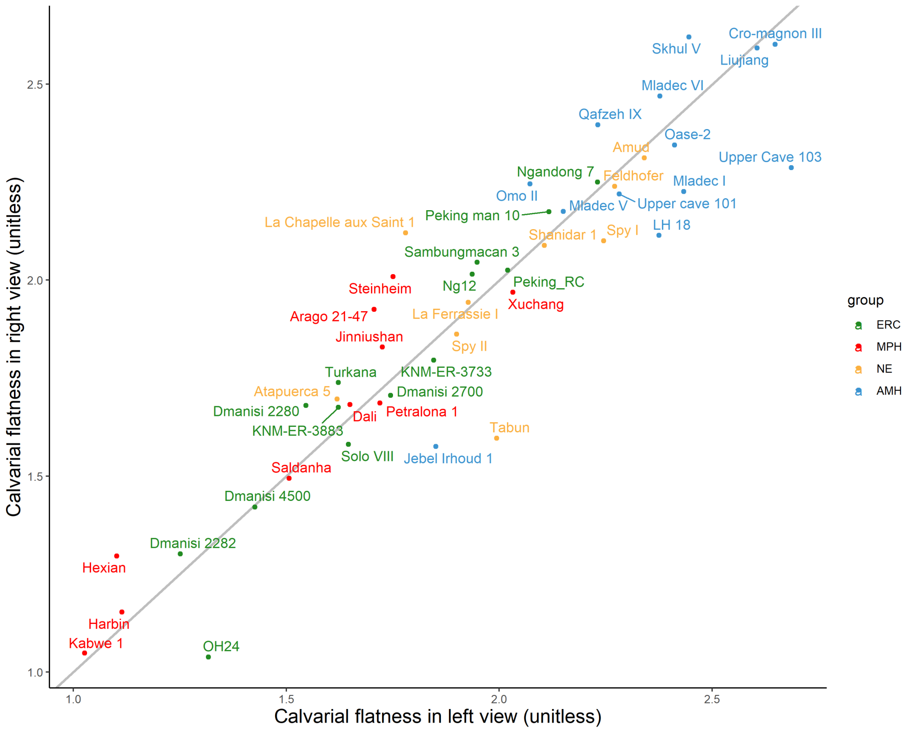

Supplementary Figure 7: Comparison of calvarial curvature values estimated by the left and right views of the crania ($R = 0.94$). Only specimens with intact contour of the calvarium are presented (blue = AMHs, yellow = Neanderthals, green = H. *erectus*, red = Middle Pleistocene specimens). Gray line depicts the curve $y = x$.

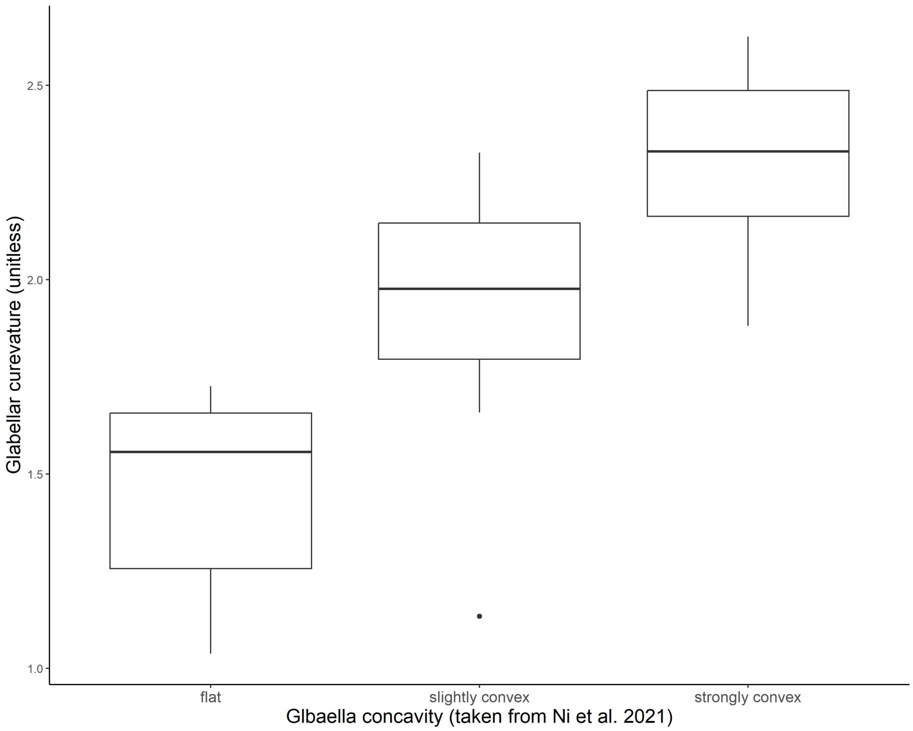

Supplementary Figure 8: Distribution of our calvarial curvature values within each of the previously described discrete classifications in Ni *et al.*[1]

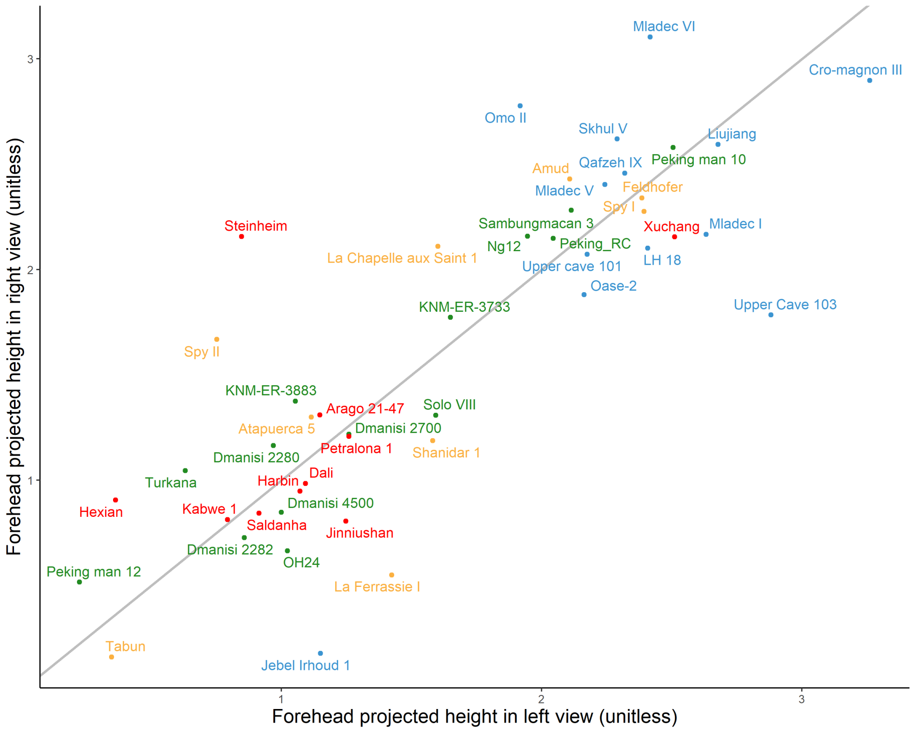

Supplementary Figure 9: Comparison of forehead height estimated by the left and right views of the crania ($R = 0.57$). Only specimens with intact contour of the calvarium are presented (blue = AMHs, yellow = Neanderthals, green = H. *erectus*, red = Middle Pleistocene specimens). Gray line depicts the curve $y = x$.

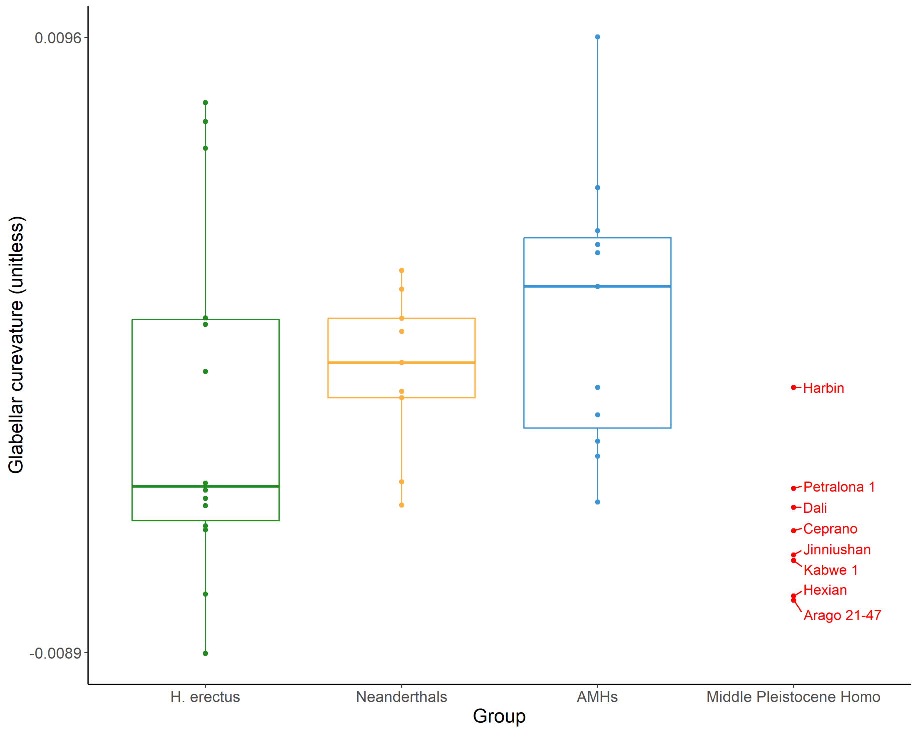

Supplementary Figure 10: Distributions of our glabellar curvature values within each of the human lineages, as well as their value in the test subjects.

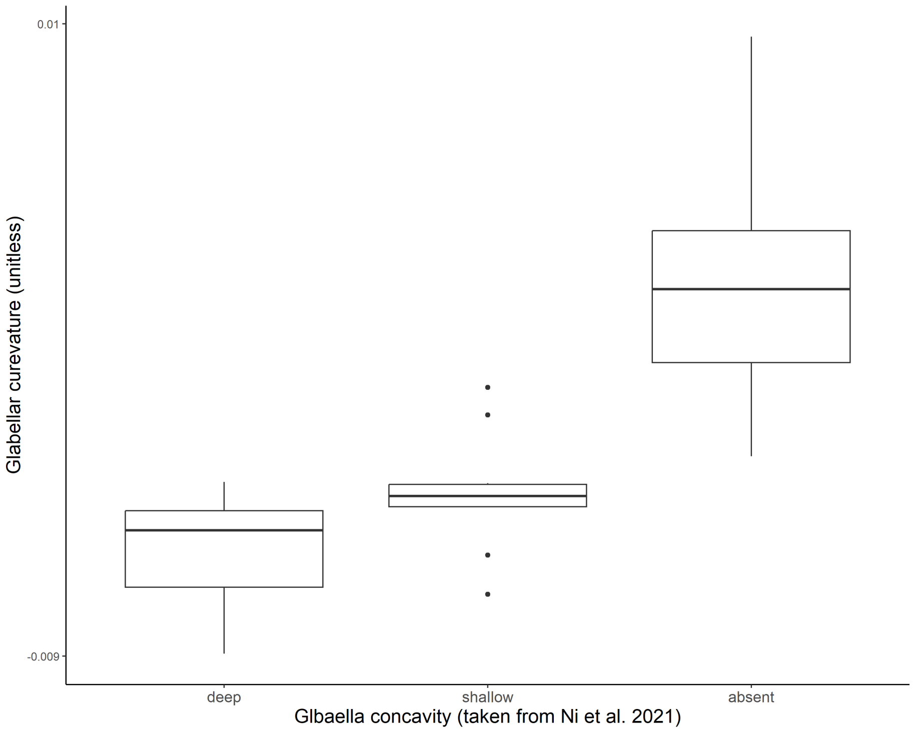

Supplementary Figure 11: Distribution of our glabellar curvature values within each of the previously described discrete classifications of Ni *et al.* [1]

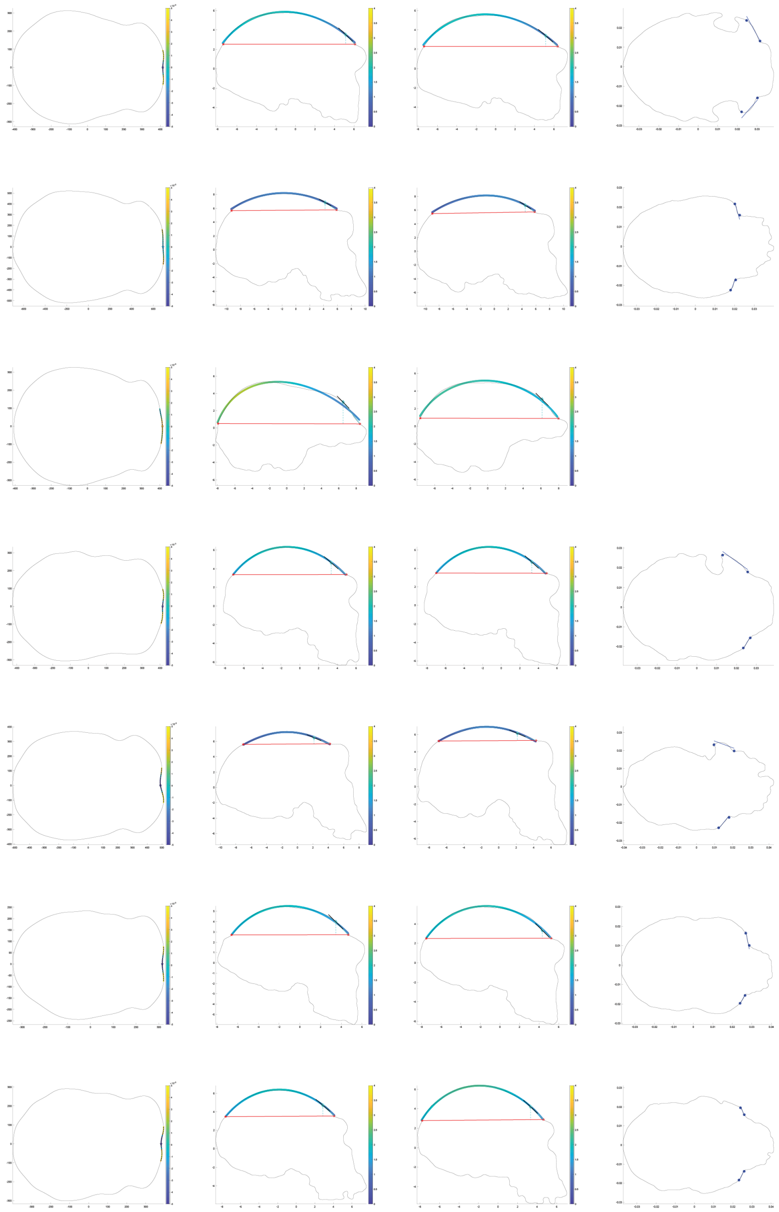

Supplementary Figure 12: Curves used to calculate calvarial curvature, forehead height, glabellar curvature and malar flattening for the test subjects. From left to right: superior view, lateral right view, horizontally flipped left lateral view and inferior view. From top to bottom: *Dali*, *Harbin*, *Xuchang 1*, *Petralona 1*, *Kabwe 1*, *Jinniushan*, *Arago 21-47*.

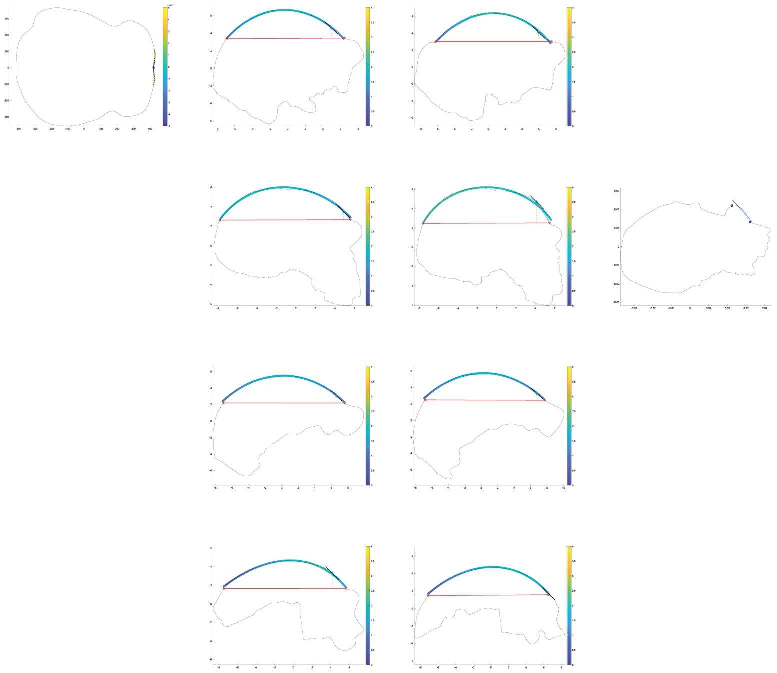

Supplementary Figure 12: (Continued) Curves used to calculate calvarial curvature, forehead height, glabellar curvature and malar flattening for the test subjects. From left to right: superior view, lateral right view, horizontally flipped left lateral view and inferior view. From top to bottom: *Ceprano*, *Steinheim*, *Saldanha*, *Maba*. Curves that were not used in the final analysis due to fragmentation or distortion are not shown.

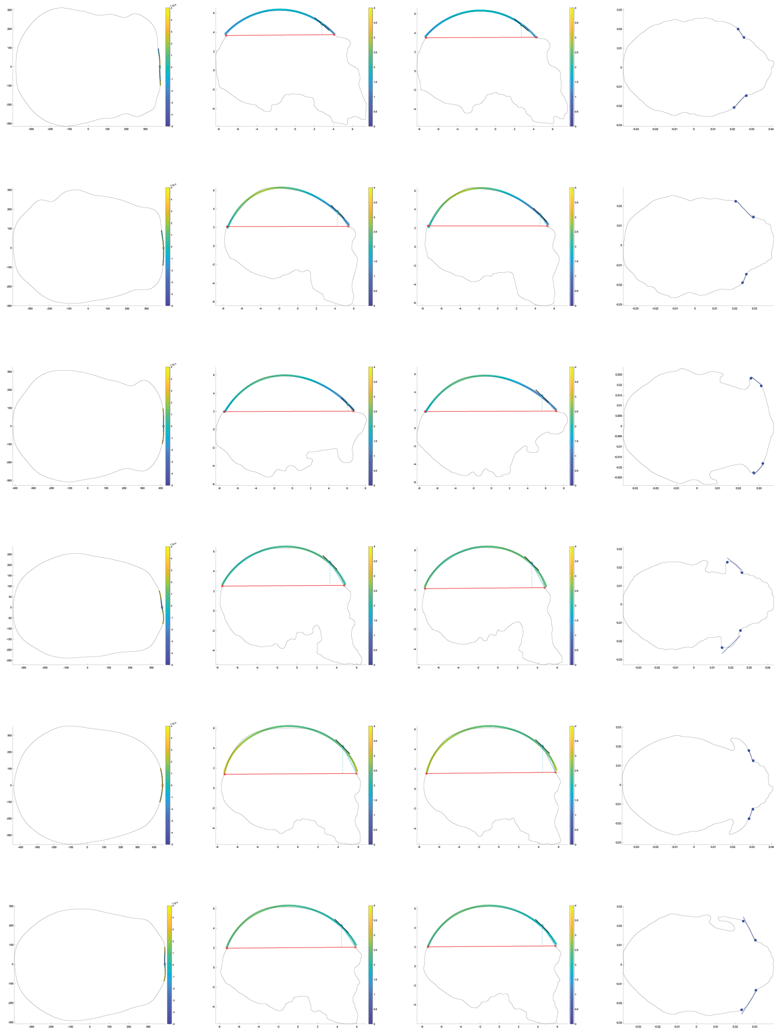

Supplementary Figure 13: Curves used to calculate calvarial curvature, forehead height, glabellar curvature and malar flattening for some of the Neanderthal and AMH specimens. From left to right: superior view, lateral right view, horizontally flipped left lateral view and inferior view. From top to bottom: *Sima de los Huesos 5*, *Shanidar 1*, *Spy II*, *Qafzeh IX*, *Liujiang*, *ZKD Upper cave 101*.

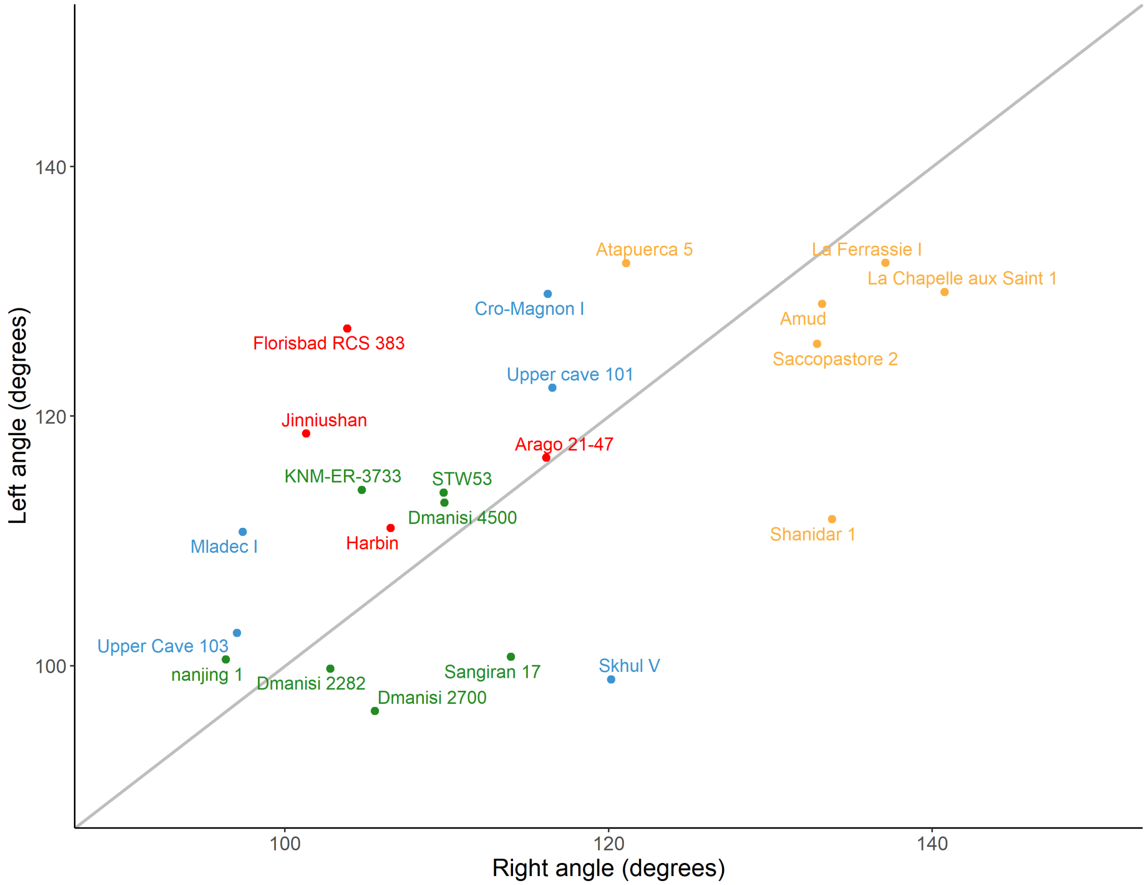

Supplementary Figure 14: Left and Right angles of specimens with correctly captured malar region. Specimens are colored based on their respective group in the analysis. Gray line depicts the curve y = x.

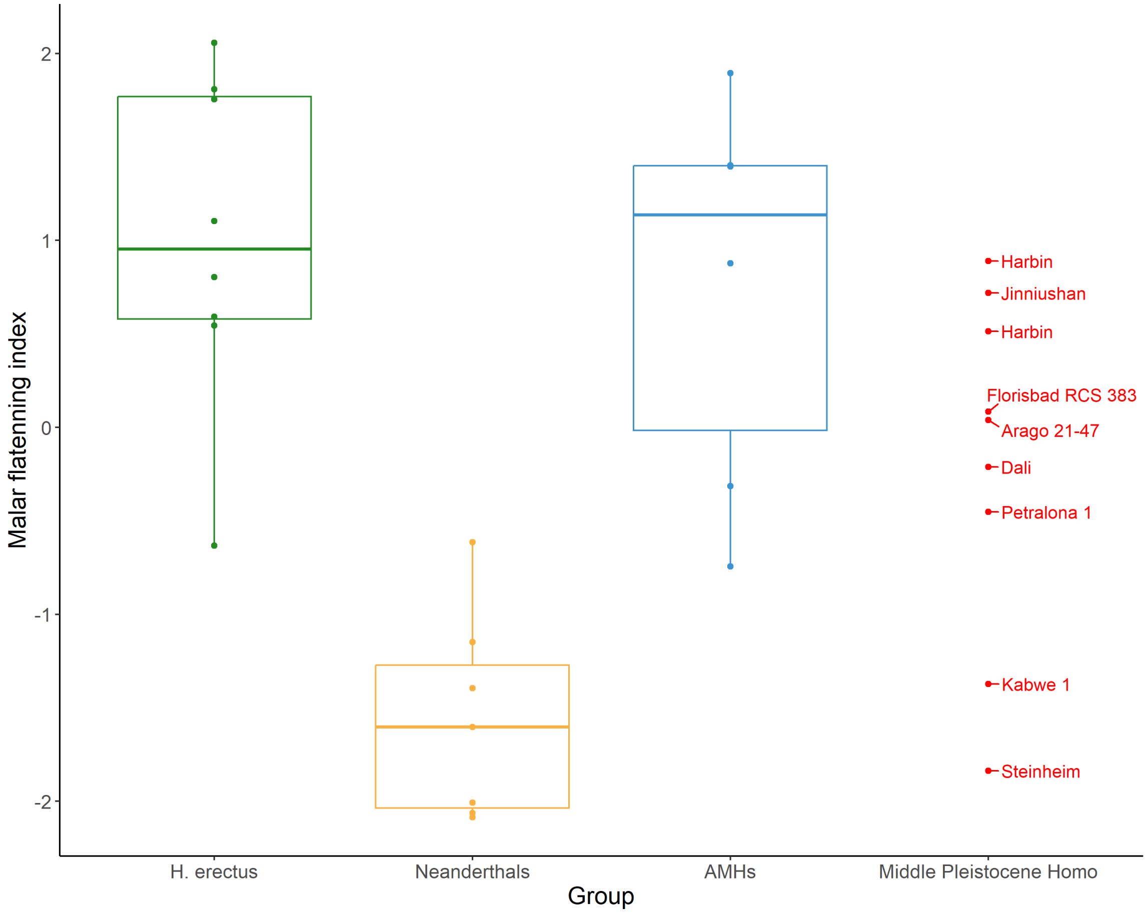

Supplementary Figure 15: Distributions of our malar index values within each of the human lineages, as well as their value in the test subjects
